# The oleocanthal-based homovanillyl sinapate as a novel c-Met inhibitor

**DOI:** 10.18632/oncotarget.8681

**Published:** 2016-04-11

**Authors:** Mohamed M. Mohyeldin, Mohamed R. Akl, Hassan Y. Ebrahim, Ana Maria Dragoi, Samantha Dykes, James A. Cardelli, Khalid A. El Sayed

**Affiliations:** ^1^ Department of Basic Pharmaceutical Sciences, School of Pharmacy, University of Louisiana at Monroe, Monroe, Louisiana, USA; ^2^ Department of Microbiology and Feist-Weiller Cancer Center, Louisiana State University Health Sciences Center, Shreveport, Louisiana, USA

**Keywords:** ABL1, breast cancer, c-Met, homovanillyl sinapate, olive oil

## Abstract

The hepatocyte growth factor (HGF)/mesenchymal-epithelial transition factor (c-Met) signaling axis has gained considerable attention as an attractive molecular target for therapeutic blockade of cancer. Inspired by the chemical structure of *S* (−)-oleocanthal, a natural secoiridoid from extra-virgin olive oil with documented anticancer activity against c-Met-dependent malignancies, the research presented herein reports on the discovery of the novel olive-derived homovanillyl sinapate (HVS) as a promising c-Met inhibitor. HVS was distinguished for its remarkable potency against wild-type c-Met and its oncogenic variant in cell-free assays and confirmed by *in silico* docking studies. Furthermore, HVS substantially impaired the c-Met-mediated growth across a broad spectrum of breast cancer cells, while similar treatment doses had no effect on the non-tumorigenic mammary epithelial cell growth. In addition, HVS caused a dose-dependent inhibition of HGF-induced, but not epidermal growth factor (EGF)-induced, cell scattering in addition to HGF-mediated migration, invasion, and 3-dimensional (3D) proliferation of tumor cell spheroids. HVS treatment effects were mediated via inhibition of ligand-mediated c-Met activation and its downstream mitogenic signaling and blocking molecular mediators involved in cellular motility across different cellular contexts. An interesting feature of HVS is its good selectivity for c-Met and Abelson murine leukemia viral oncogene homolog 1 (ABL1) when profiled against a panel of kinases. Docking studies revealed interactions likely to impart high dual affinity for both ABL1 and c-Met kinases. HVS markedly reduced tumor growth, showed excellent pharmacodynamics, and suppressed cell proliferation and microvessel density in an orthotopic model of triple negative breast cancer. Collectively, the present findings suggested that the oleocanthal-based HVS is a promising c-Met inhibitor lead entity with excellent therapeutic potential to control malignancies with aberrant c-Met activity.

## INTRODUCTION

Mesenchymal-epithelial transition factor (c-Met) is the prototypic member of a unique subfamily of receptor tyrosine kinases (RTKs) and its high-affinity natural ligand is hepatocyte growth factor (HGF) [1]. c-Met and HGF are required for normal cellular development and homeostasis. HGF stimulation triggers c-Met induction of several biological responses that collectively give rise to invasive growth during embryo development [2, 3]. In adults, c-Met mediated invasive growth becomes quiescent; it is normally only fully active during wounds healing and tissues regeneration [1, 4]. However, the HGF/c-Met axis is frequently reactivated by cancer cells for tumorigenesis, invasive growth, and metastatic progression [5]. A broad range of mechanisms may lead to anomalous c-Met signaling, including protein overexpression, gene amplification, activating gene mutations, increased paracrine stimulation and acquisition of autocrine signaling [1]. There is a mounting evidence for the involvement of dysregulated activation of c-Met signaling in the development and progression of multiple types of cancers, inducing cell proliferation, angiogenesis, and survival [2]. Aberrant activation of the HGF/c-Met axis is known to promote cytoskeletal changes of many cancer cells, inducing migration, invasion, and eventual metastasis [6–8]. In addition, phosphorylated c-Met has been considered as an important predictor of tumor aggressiveness, metastatic potential and poor survival [4]. Activation of c-Met signaling and/or HGF up-regulation in the tumor microenvironment may confer resistance to RTKs-targeting cancer therapies already in clinical use, including epidermal growth factor receptor (EGFR) and v-RAF murine sarcoma viral oncogene homolog B1 (BRAF) kinase inhibitors. This mainly due to the ability of c-Met to cross-talk with a variety of cell surface receptors [9, 10]. Therefore, inhibition of the HGF/c-Met signaling axis has a great potential for therapeutic intervention in cancer.

To date, the main strategies employed to interrupt c-Met signaling involve blocking the interaction between c-Met and HGF via neutralizing antibodies directed against either HGF or c-Met, or truncated forms of HGF with antagonistic activity on c-Met such as NK4, or c-Met biologics such as ribozymes, dominant-negative receptors, decoy receptors, and peptides [11]. The latter approaches, by definition, do not address ligand-independent c-Met activation and may only be effective for HGF-driven cancers. Recent efforts have focused on interfering with the active site of the kinase domain with small-molecule kinase inhibitors [12, 13]. Whereas most of former approaches are better suited to block ligand-mediated c-Met activity, small-molecule c-Met inhibitors offer the most versatile approach by inhibiting HGF-dependent cancers as well as tumors driven by other Met-dependent mechanisms, such as receptor amplification and activating mutations [14]. Therefore, targeting the c-Met kinase with small-molecule inhibitors can be considered a promising approach for cancer treatment and prevention, with respectable number of these small molecules in clinical trials. However, current FDA-approved c-Met inhibitors include only crizotinib (Xalkori; Pfizer) and cabozantinib (Cometriq; Exelixis) for the treatment of late-stage non-small cell lung cancer (NSCLC) and progressive metastatic medullary thyroid cancer, respectively [12]. Both compounds are non-specific c-Met inhibitors, such that crizotinib also targets v-ros avian UR2 sarcoma virus oncogene homolog 1 (ROS1) and anaplastic lymphoma kinase (ALK) while cabozantinib inhibits vascular endothelial growth factor receptor 2 (VEGFR2), in addition to c-Met. Cancer patients frequently become resistant to c-Met inhibitors, mandating a continued need to discover additional small-molecule inhibitors of the c-Met pathway.

**Figure 1:**
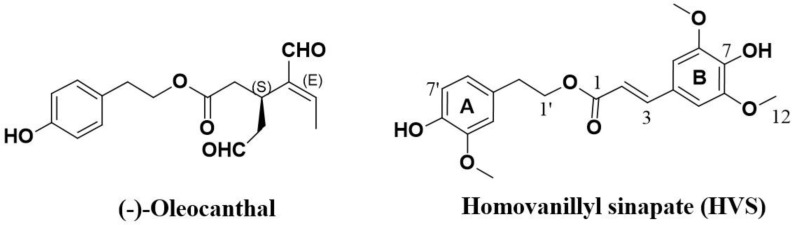
Chemical structures of *S* (−)-oleocanthal and homovanillyl sinapate (HVS)

Epidemiological and clinical studies have compellingly suggested that the incidence of particular types of cancer in Mediterranean countries is lower than in other populations. This may be attributed to the Mediterranean dietary regimen rich in extra-virgin olive oil (EVOO), apart from possible genetic factors [15]. *S* (−)- Oleocanthal (Figure 1), a naturally occurring secoiridoid from EVOO, has attracted considerable attention due to its various biological effects against inflammation, Alzheimer's disease, and cancer [16–18]. Oleocanthal has been shown to mediate its anticancer effects through the disruption of c-Met related pathways [16, 19]. Recently, the intracellular mechanisms of oleocanthal and its c-Met receptor signaling suppression have been characterized in breast cancer mouse model, promoting this unique natural product from the hit to the lead rank [19].

In continuation of interest in pursuing novel therapeutically useful c-Met inhibitors, a series of semisynthetic optimization driven by the chemical structure of oleocanthal and *in silico* studies resulted in the discovery of a novel oleocanthal-based c-Met inhibitor hit named homovanillyl sinapate (HVS, Figure 1). Chemically, the structure of HVS is unique with its homovanillyl alcohol and sinapic acid parent components, which naturally occur in olive (Figure 1). The present study deals with the hit-to-lead promotion of this oleocanthal-based HVS as a novel small-molecule c-Met inhibitor. The study aims at characterization of the intracellular mechanisms involved in mediating the anticancer effects of HVS and the potential involvement of c-Met receptor signaling. HVS is believed to serve as an excellent template or scaffold for the development of structurally similar and more efficacious anti-c-Met therapeutic agents.

## RESULTS

### HVS potently inhibited the catalytic activity of c-Met and its oncogenic variant *in vitro*

**Figure 2:**
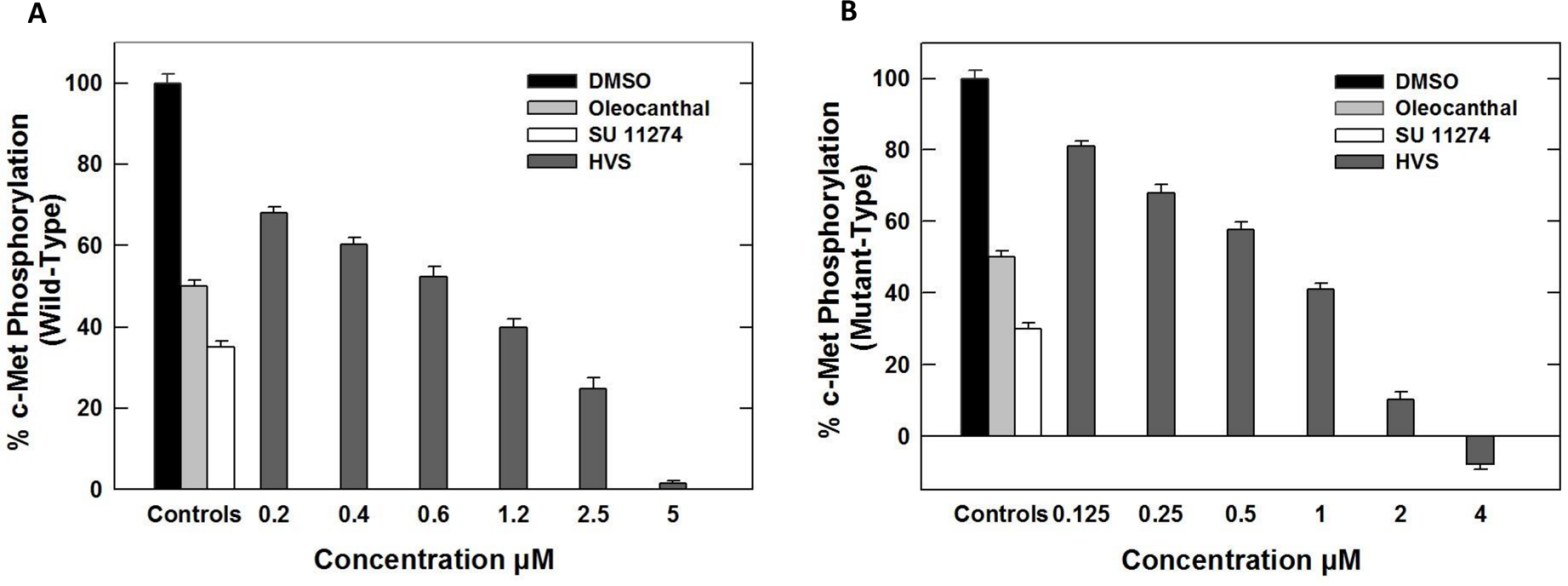
(**A**) Effect of HVS on the phosphorylation (activation) of wild-type recombinant human c-Met kinase at different concentrations, using Z’-LYTE assay kit. (**B**) Effect of HVS on the phosphorylation (activation) of mutant-type recombinant human c-Met kinase (M1250T) at different concentrations, using Z’-LYTE assay kit. Error bars indicate the SEM of *n* = 3/dose; SU11274 and (−)-oleocanthal were used as positive controls at 1 and 5 μM, respectively [16, 34].

HVS (Figure 1) was selected for further characterization based on screens designed to identify c-Met inhibitors. The c-Met-mediated functions are dependent on its kinase activity and therefore the *in vitro* ability of HVS to inhibit c-Met phosphorylation (activation) was directly tested on the purified kinase domain of c-Met (amino acids 956–1390) that was *in vitro* phosphorylated to achieve the highest level of intrinsic kinase activity [14]. In this experiment, Z′-LYTE™ Tyr6 peptide was used as a substrate; thus, the changes in its phosphorylation can directly reflect the c-Met kinase activity. Meanwhile, *S* (−)-oleocanthal and the standard c-Met competitive inhibitor SU11274 were used as positive controls for activity comparison. The calculated IC_50_ of *S* (−)-oleocanthal in this assay was 5.2 μM (Table 1), which was consistent with its reported IC_50_ value (4.8 μM), validating this study results [16]. HVS was shown to be a potent inhibitor of recombinant wild-type c-Met kinase in this cell-free assay, inhibiting c-Met phosphorylation induced by the addition of ATP in a dose-dependent manner, with an IC_50_ of 1 μM, and demonstrating nearly five-fold activity improvement compared to *S* (−)-oleocanthal (Figure 2A, Table 1).

**Table 1:** IC_50_ values for HVS in different functional assays used throughout the study

Assay/Cell Line or Kinase	HVS	(−)-Oleocanthal^a^
	IC_50_ (μM) ± SEM	
Z’-LYTE^™^ kinase/Wild-type c-Met	1.0 ± 0.2	5.2 ± 0.8
Z’-LYTE^™^ kinase/Mutant-type c-Met (M1250T)	0.9 ± 0.4	3.9 ± 1.2
Z’-LYTE^™^ kinase/Mutant-type c-Met (Y1230C)	> 10	> 10
Z’-LYTE^™^ kinase/Mutant-type c-Met (Y1235D)	> 10	> 10
MTT proliferation/MDA-MB-231	3.8 ± 0.8	12.2 ± 1.4
MTT proliferation/MDA-MB-468	6.0 ± 0.5	15.4 ± 2.8
MTT proliferation/MCF-7	8.7 ± 0.9	22.7 ± 1.1
MTT proliferation/BT-474	12.2 ± 0.7	27.2 ± 2.1
MTT proliferation/T-47D	> 40	35.4 ± 0.7
Wound-healing migration/MDA-MB-231	2.5 ± 0.6	8.5 ± 1.1
Cultrex^®^ BME cell invasion/MDA-MB-231	2.7 ± 0.8	18.2 ± 1.6

a The IC_50_ values of *S*-oleocanthal have been included for activity comparison.

Several c-Met-activating mutations have been identified in numerous human cancers [20]. Early identification of new hit abilities to inhibit wild-type and mutant kinases is essential for subsequent drug development process to design drugs useful for patients harboring c-Met mutations [20]. HVS was evaluated for its ability to inhibit c-Met phosphorylation across three c-Met mutant variants, including two activation loop mutants Y1230C and Y1235D, as well as the P+1 loop mutant M1250T, which is near the ATP binding site. Selection of these well-characterized mutations was based on the ability of M1250T mutant to display the strongest kinase activity and the highest neoplastic transforming potential among all c-Met mutants. Meanwhile the activation loop missense mutations reportedly confer absolute or partial resistance to several known c-Met inhibitors [14, 21, 22]. In presence of 200 μM ATP, HVS exhibited slightly improved activity against M1250T oncogenic human c-Met mutant, with IC_50_ value of 0.9 μM, compared with the wild-type c-Met (Figure 2B, Table 1). In contrast, a marked shift in HVS potency was observed against c-Met activation loop mutant variants Y1230C and Y1235D (IC_50_ > 10 μM, Table 1) compared with wild-type receptor.

### *In silico* binding mode analyses of HVS in wild and mutant c-Met crystal structures

**Figure 3:**
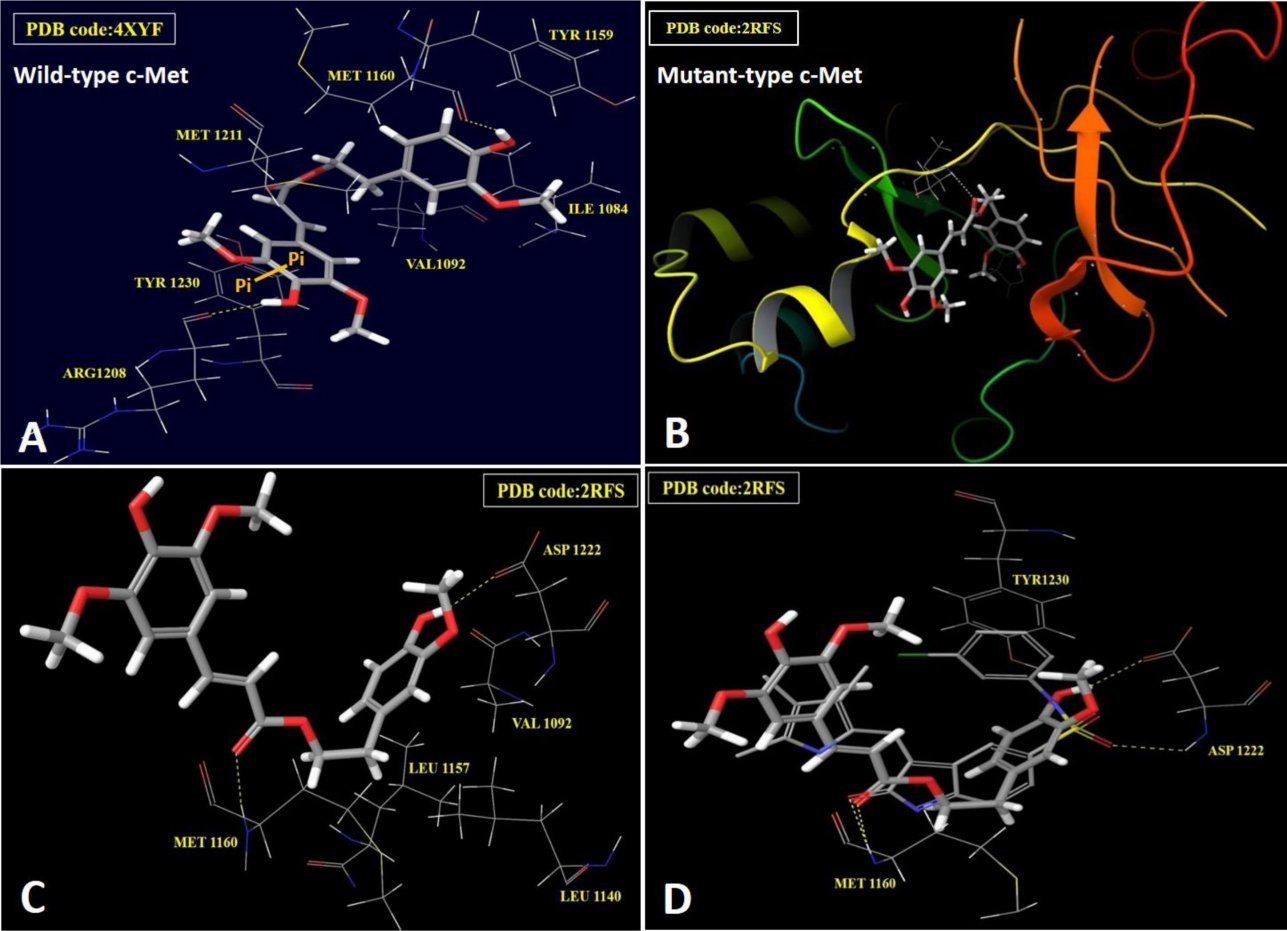
*In silico* binding mode of HVS at the ATP binding site of wild and mutant-types of c-Met kinase domain (**A**) Important interactions of HVS at the ATP binding site of wild-type c-Met (PDB code: 4XYF). (**B**) Overview of HVS's binding pose at the ATP binding site of mutant-type c-Met (PDB code: 2RFS). (**C**) Important interactions of HVS at the ATP binding site of mutant-type c-Met (PDB code: 2RFS). (**D**) Structure overlay for HVS (shown in tube) with the original ligand SU11274 conformations (shown in thin tube) obtained from the crystal structure of mutant-type c-Met (PDB code: 2RFS) and docking simulation.

The differential activity of HVS against c-Met oncogenic variants in cell-free assays encouraged the *in silico* docking study using the highly resolved co-crystal structure of SU11274 with c-Met kinase (PDB code: 2RFS) as a docking model, in an attempt to elucidate HVS's binding mode within mutant-type c-Met and compare it with its pose in the ATP binding site of the wild-type (PDB code: 4XYF, Figure 3). Selection of 2RFS crystal structure was based upon a previous study reporting this crystal structure for c-Met protein as the one containing the nine known activating point mutations in the c-Met proto-oncogene, including M1250T [14]. HVS showed good fitting within the ATP binding pocket of mutant-type c-Met (Figure 3B). The HVS model conformation is roughly C-shaped and wraps around the Met1211 of the c-Met kinase domain's activation loop. Surprisingly, HVS binds 10 Å away from the C-helix pocket with a dramatic difference in its binding mode in the mutant-type compared to the wild-type c-Met (Figure 3A). Perhaps the most interesting and unexpected feature of the structure of HVS bound to the mutant-type c-Met is the absence of any binding role for the sinapic acid moiety at the ATP binding pocket (Figure 3C). Alternatively, the phenolic hydroxyl group on the homovanillyl aromatic ring A as well as the carbonyl group of the ester functionality in HVS presented the main binding and anchoring pharmacophoric groups at the mutant c-Met kinase domain (Figure 3C). The ester carbonyl group of HVS is participating in a critical hydrogen bonding (HB) interaction with the backbone NH of the Met1160 at the hinge region of the mutant c-Met kinase, unlike the wild-type where the ester moiety appeared not to contribute any binding role at the ATP binding pocket (Figure 3A and 3C). This unique HB interaction appeared to stabilize the C-shaped conformation of HVS within the pocket of the mutant-type which in turn, allowed an exceptional fitting of the terminal ring A along with its 2-ethyl side chain in a deep hydrophobic pocket composed of Ala1226, Leu1157, Ala1108, Leu1140 and Val1092, making predominantly hydrophobic interactions with the protein and mimicking the indolin-2-one core group of SU11274 (Figure 3D). Additionally, the phenolic hydroxyl group on ring A was uniquely engaged in a HB interaction with the side chain carbonyl group of Asp1222 at the activation loop (Figure 3C). This interaction caused c-Met to adopt a unique inactive conformation in its complex with HVS. In particular, Asp1222 is rotated away from the active site where it is unable to coordinate Mg^2+^ and assist in catalysis. The mutation M1250T lies in the large kinase domain about 18 Å away from HVS. This may justify the little impact of this mutation on HVS's c-Met inhibitory potency. The activating mutations Y1230C and Y1235D, which have been shown to confer resistance to HVS, are both located in the activation loop, which is disordered in this crystal structure and that is why it is difficult to rationalize why HVS was not able to inhibit these mutations equally well as wild-type c-Met. A possible explanation for the lack of c-Met inhibitory potential against Y1230C mutant could be that HVS was not able to form HB interactions with the backbone of Tyr1230, rather than simply making an aromatic ring stacking interaction, via ring B in HVS, with its side chain in the wild-type c-Met (Figure 3A). Both cell-free and docking results rendered HVS a potential c-Met inhibitor hit appropriate for further validation.

### Effect of HVS on cancer cell proliferation

**Figure 4:**
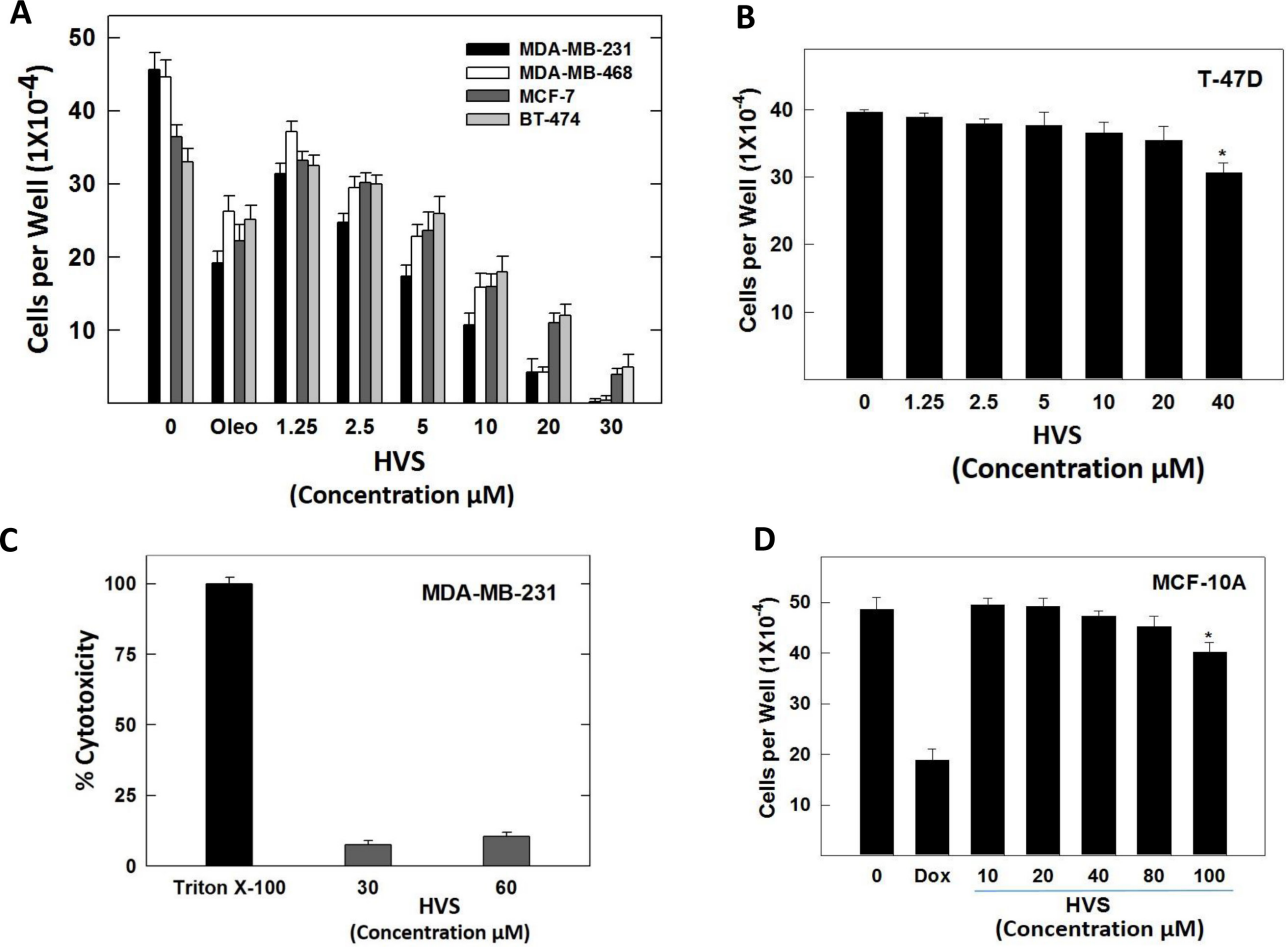
(**A**) Effect of HVS treatment on HGF-stimulated growth of Met-dependent MDA-MB-231, MDA-MB-468, MCF-7, and BT-474 breast cancer cells after 72 h treatment period, compared to DMSO as vehicle control. Viable cell count was determined using MTT assay. Vertical bars indicate the mean cell count ± SEM of *n* = 3 in each treatment group; (−)-oleocanthal was used as a positive control at 20 μM [19]. (**B**) Effect of HVS treatment on HGF-stimulated growth of Met-independent T-47D breast cancer cells after 72 h treatment period. Viable cell count was determined using MTT assay. Vertical bars indicate the mean cell count ± SEM of *n* = 3 in each treatment group. (**C**) Cytotoxic effects of HVS on the highly metastatic MDA-MB-231 breast cancer cells at two different concentrations (30 and 60 μM) using LDH cytotoxicity assay. Triton X-100 was used as a positive control to cause the breakdown of all the cells and induce the total possible LDH release. (**D**) Effects of HVS treatment on the viability of non-tumorigenic human MCF-10A mammary epithelial cells after a 24 h treatment period, compared to DMSO as a vehicle control. Viable cell count was determined using MTT assay. Vertical bars indicate the mean cell count ± SEM of *n* = 3 in each treatment group; doxorubicin was used as a positive control at 10 μM dose. *P < 0.05 as compared with a vehicle-treated control.

HVS was further evaluated in a series of cell-based functional assays to confirm the cell-free and docking results. The activation of c-Met with HGF is known to play an important role in cell proliferation in many kinds of cancer cells. Therefore, the aim of this part was to analyze the antiproliferative effects of HVS on key human breast cancer cell lines using the MTT assay. The cell lines were chosen to represent a wide range of breast cancer phenotypes. ERα, one of the most important targets in human breast cancer therapy, is expressed in MCF-7, BT-474 and T-47D cells, whereas MDA-MB-231 and MDA-MB-468 cells lack the expression of ERα due to epigenetic silencing [23]. c-Met is expressed at higher levels in MDA-MB-231 and MDA-MB-468 cells, and expressed at relatively lower levels in MCF-7 and BT-474 cells, while it is absent in T-47D cells [24, 25]. Accordingly, the four human breast cancer cell lines expressing c-Met, namely MBA-MD-231, MDA-MB-468, MCF-7, and BT-474, were chosen to determine the antiproliferative activities of HVS. Additionally, T-47D cells have been used to evaluate the off-target effects of HVS in a c-Met-independent breast cancer model. HGF at a concentration of 40 ng/mL was used as mitogen to induce c-Met-dependent effects in breast cancer cells, according to optimization studies previously described [19]. (−)-Oleocanthal with defined c-Met inhibitory activity was used as a standard positive control [19]. The antiproliferative effects of various doses of HVS on HGF-mediated growth of MDA-MB-231, MDA-MB-468, MCF-7, and BT-474 breast cancer cell lines after 72 h culture period are shown in Figure 4A. Treatment with HVS caused a dose-dependent suppression of HGF-induced proliferation of the four cell lines (Figure 4A). In general, HVS was more potent against MDA-MB-231 and MDA-MB-468 compared to the other two cell lines. Interestingly, larger concentrations of HVS were required to significantly abolish the cell viability of MDA-MB-231, MDA-MB-468, MCF-7 and BT-474 cells in HGF-free media after 72 h (Figure S3). The IC_50_ values for HVS treatment in HGF-supplemented media were 3.8, 6.0, 8.7 and 12.2 μM in MDA-MB-231, MDA-MB-468, MCF-7, and BT-474 cells, respectively (Figure 4A, Table 1). However, the IC_50_ values for HVS in HGF-free media were > 40 μM in the four breast cancer cell lines (Figure S3). Importantly, HVS lacked activity against the Met-independent T-47D breast cancer cell line (IC_50_ > 40 μM, Figure 4B, Table 1). These results clearly indicated that HVS specifically inhibited the HGF-dependent growth across multiple c-Met-expressing breast cancer cell lines in a dose-responsive manner as compared to their vehicle-treated control groups (Figure 4A). It was interesting to note that the dose-dependent loss of proliferative capacity due to HVS treatment positively correlates with the level of c-Met expression in different human breast cancer cells, supporting the hypothesis that c-Met inhibition can be the mechanism for the antitumor effects of this oleocanthal-based derivative. The activity of HVS was several folds that of the parent positive control, oleocanthal, in all investigated cancer cell lines indicating its potential as a novel antiproliferative entity (Figure 4A, Table 1).

### Effect of HVS on lactate dehydrogenase (LDH) release in cancer cells

Tetrazolium-based assays may overestimate the effectiveness of a drug that alters cell metabolism but not cell viability due to the fact that such assays measure cellular metabolism as indirect readout for cell proliferation [26]. These assays also failed to differentiate between cell cycle inhibition and cellular death [27]. To overcome these problems, the MTT assay was multiplexed with the LDH-based cytotoxicity assay to allow further quantification by determining the percentage of dead cells after HVS treatment and distinguish whether HVS causes cancer cell death or growth arrest without the need of specialized equipment [27]. In this assay, the standard protocol bases the total possible LDH release on a single control where Triton X-100 is added to cause the breakdown of all cells and the supernatant from these wells is then used as a reference for the total possible amount of LDH. The cytotoxicity assay was performed using MDA-MB-231 breast cancer cells, cultured with 2 different doses of HVS (30 and 60 μM), in the presence of 40 ng/ mL HGF, to induce cell death and LDH release. HVS treatment showed 7.5 and 10.4% cytotoxicity at 30 and 60 μM doses, respectively, indicating that HVS did not exhibit a significant cytotoxicity up to 16-folds higher than its IC_50_ in MDA-MB-231 cells (Figure 4C). Cancer cells with ligand-dependent c-Met activity, e.g., MDA-MB-231 cells, underwent cytostasis and were less susceptible to apoptosis induction following HVS treatment.

### Effect of HVS on non-tumorigenic human mammary epithelial cell growth

To evaluate the relative selectivity of HVS towards malignant cells, the immortalized non-tumorigenic human MCF-10A mammary epithelial cells were treated with various concentrations of HVS for 24 h. Treatment with 10–80 μM of HVS had no effect on MCF-10A cells viability as compared to the vehicle treated control group (Figure 4D). In contrast, a 10 μM of the cytotoxic doxorubicin resulted in 60% reduction in the MCF-10A cells viability after 24 h (Figure 4D). These results suggested the selectivity of HVS antiproliferative effects toward breast cancer cells.

### Effect of HVS on cancer cells migration

**Figure 5:**
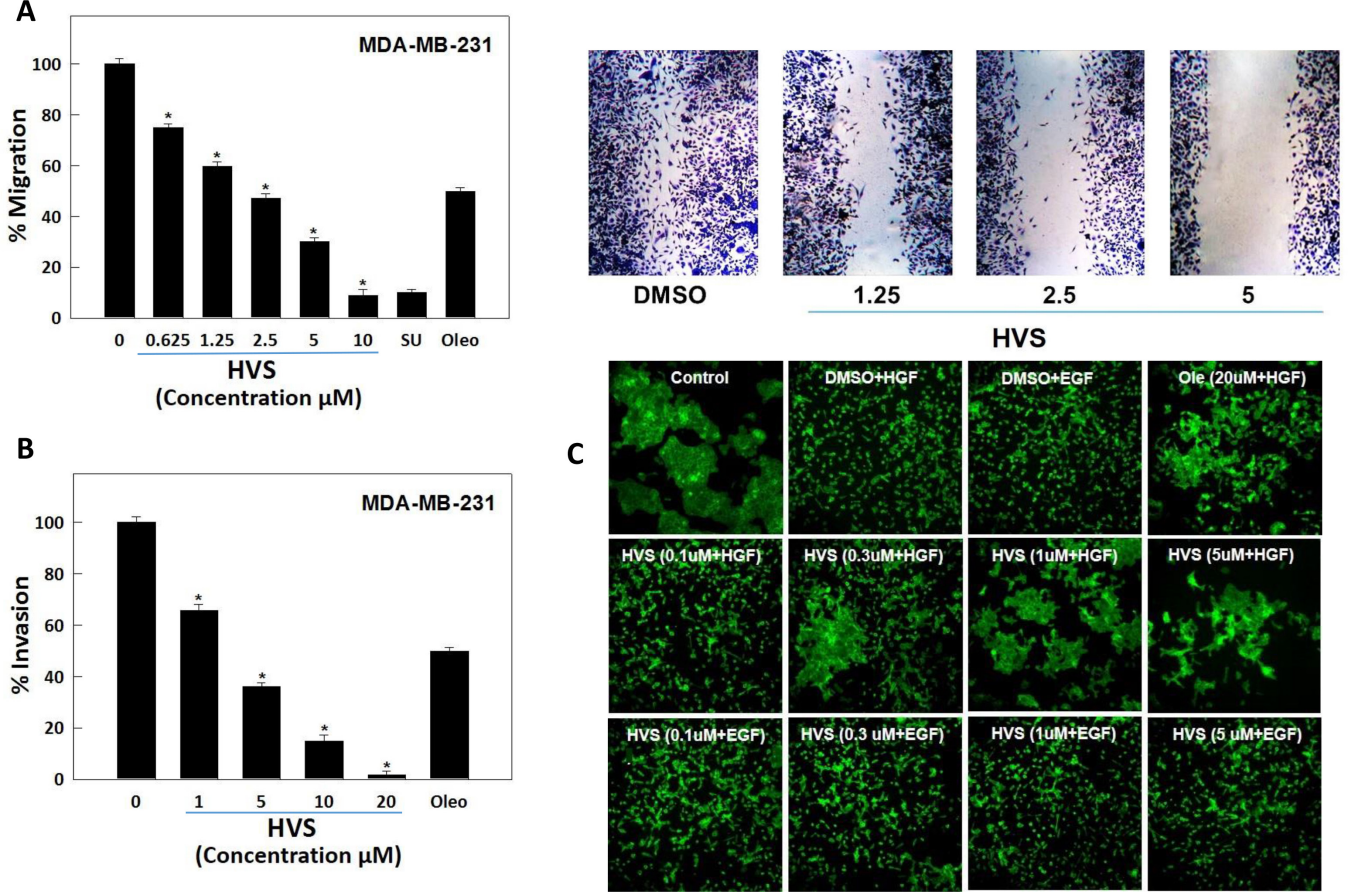
(**A**) Effect of HVS on HGF-induced migration of the highly metastatic MDA-MB-231 human breast cancer cells in wound healing assay. *Left panel* shows quantitative analysis of the percentage of gap reduction (i.e., wound closure) in various treatment groups in MDA-MB-231 cancer cells. Vertical bars indicate the percentage of wound closure at 24 h after wounding, calculated relative to the wound distance at zero time ± SEM of *n* = 6 in each treatment group. SU11274 and (−)-oleocanthal were used as positive controls at 10 μM [19, 34]. *Right panel* represents photomicrographs of wound healing assay showing HVS treatment at three different concentrations (1.25, 2.5, and 5 μM) blocked the migration of MDA-MB-231 cells in response to HGF stimulation, as compared to DMSO as a vehicle control. (**B**) Effect of HVS on HGF-induced cell invasion of the highly aggressive MDA-MB-231 human breast cancer cells using Cultrex^®^ BME cell invasion assay. The cells were treated with different concentrations of HVS for 24 h. Vertical bars indicate the percentage of cells invading the basement membrane at the end of treatment period ± SEM of *n* = 3 in each treatment group. (−)-Oleocanthal was used as a positive control at 20 μM dose [34]. *P < 0.05 as compared with vehicle-treated control. (**C**) Effect of HVS on HGF-induced scattering of the DU145 human prostate cancer cells in 2D monolayer cultures, compared to DMSO as vehicle control. Cells were treated with indicated concentrations of HVS for 30 minutes in serum-free media. 33 ng/mL HGF or 100 ng/mL EGF was spiked into the appropriate wells and cells were allowed to scatter for 16 h. Cells were fixed and stained with phalloidin. Representative 10X images are shown, *n* = 3. (−)-Oleocanthal was used as a positive control at 20 μM dose.

In addition to the modulation of cell proliferation, activation of the HGF/c-Met axis promotes cell migration and invasion, which contribute to the metastatic characteristics of malignant cells [28]. The wound healing assay is a simple method to assess directional cell migration *in vitro* [19]. Thus, HVS was evaluated for its antimigratory effect in the scratch assay using the highly metastatic MDA-MB-231 cells. HGF (40 ng/mL) was used in the growth media as a mitogen to induce cell migration via c-Met activation. HVS significantly inhibited cell migration across the wound inflicted in the MDA-MB-231 cells monolayer compared to the vehicle control and a 10 μM dose of *S* (−)-oleocanthal and SU11274 as positive controls (Figure 5A) [19]. Apparently, HVS treatment significantly suppressed HGF-induced mammary tumor cell migration in a dose-dependent manner, with an IC_50_ value of 2.5 μM, demonstrating three-fold improvement versus its parent (−)-oleocanthal (IC_50_ 8.5 μM, Figure 5A, Table 1).

### Effect of HVS on cancer cells invasion

MDA-MB-231 breast cancer cell line was used in the invasion assay for its c-Met-dependent aggressive and highly invasive nature [23]. The effect of HVS on cell invasion was examined using the Cultrex^®^ basement membrane extract (BME) cell invasion assay. HGF (40 ng/mL) was used in the growth media as a mitogen to induce cell invasion. HVS treatment for 24 h significantly decreased the level of HGF-mediated MDA-MB-231 cells invasion through the matrigel in a dose-dependent manner compared to the vehicle-treated cells, with an IC_50_ of 2.7 μM, demonstrating seven-fold activity enhancement over the parent oleocanthal (IC_50_ 18.2 μM, Figure 5B, Table 1).

### Effect of HVS on cancer cells scattering

Activated HGF/c-Met signaling is known to promote cell scattering which stimulates cells to abandon their original environment, a hallmark of cancer invasiveness and metastasis [28]. It has been well documented that DU145 prostate cancer cells, which normally grow in clusters, exhibit a disruption and scattering of the cell colonies upon HGF stimulation. Thus, the effect of HVS on HGF-stimulated DU145 cells scattering was studied. Additionally, DU145 cells were stimulated with epidermal growth factor (EGF) to evaluate the off-target effects of the same treatment doses of HVS in this *in vitro* model. Cells were treated with increasing doses (0.1, 0.3, 1, and 5 μM) of HVS or (−)-oleocanthal at 20 μM for activity comparison. Cells were then stimulated with either HGF or EGF for 16 h. Finally, cells were fixed and stained with phalloidin (Figure 5C). Immunofluorescence microscopy revealed that HVS treatment potently reduced HGF-mediated scattering of DU145 cells in a dose-dependent manner, completely blocking cell spreading at concentrations as low as 0.3 μM (Figure 5C). HVS has demonstrated more than twenty-fold enhancement in the activity versus *S* (−)-oleocanthal which partially prevented HGF-mediated cell scattering at 20 μM, providing a clear evidence of HVS's superior activity over (−)-oleocanthal across different cellular contexts. Interestingly, HVS did not affect the EGF-mediated scattering in prostate cancer cells, confirming that this oleocanthal-based derivative significantly impaired tumor cell motility and invasiveness mediated only by the HGF/c-Met axis and eliminated the possibility of off-target effects (Figure 5C). Overall, HVS proved a robust inhibitor of HGF-induced scattering in DU145 prostate cancer cells.

### Effect of HVS on three-dimensional (3D) spheroid growth in breast and prostate cancer cells

**Figure 6:**
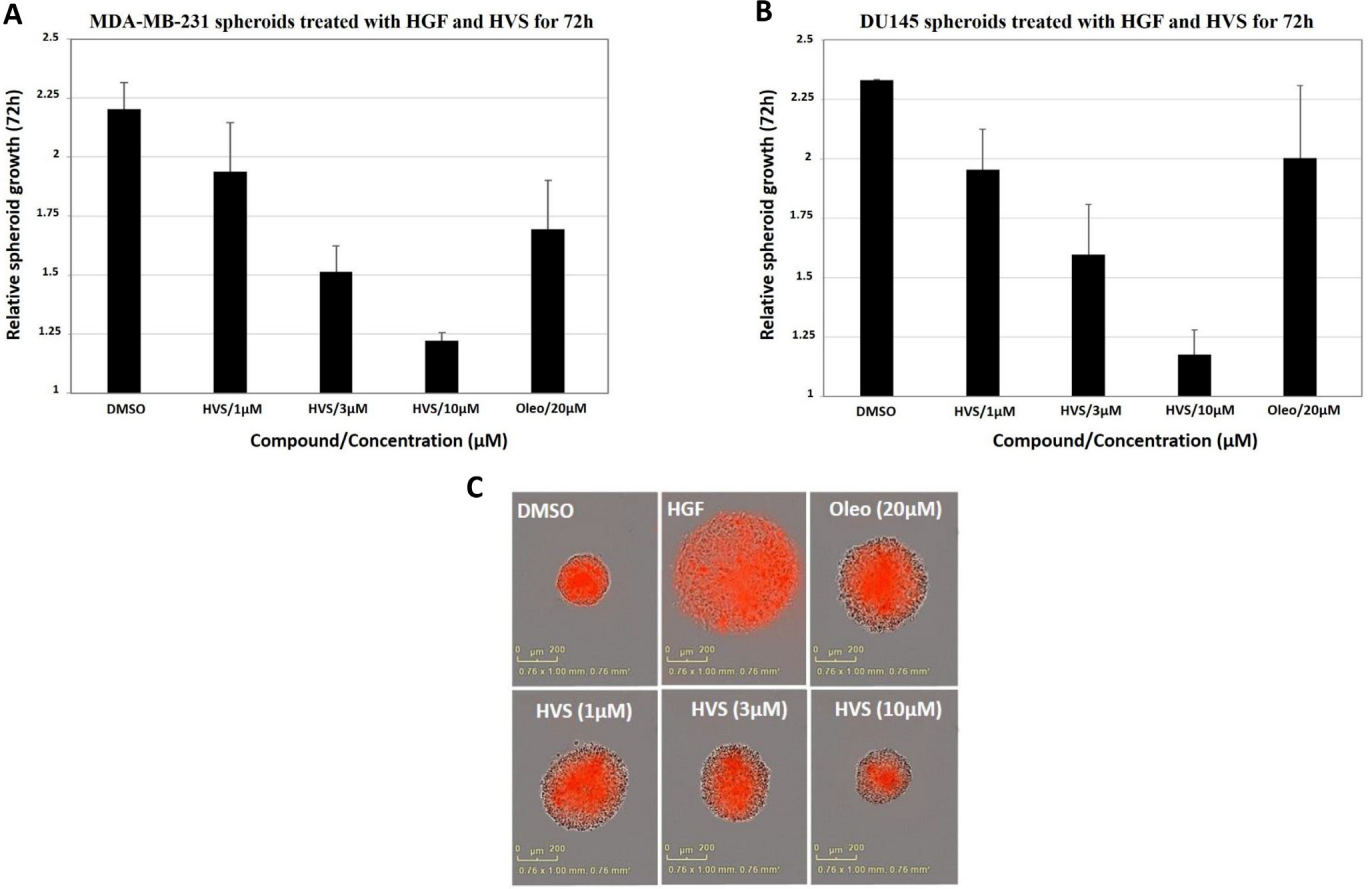
Effect of HVS on the HGF-induced 3D spheroid growth of (A) the human breast MDA-MB-231 cancer cells and (B) the human prostate DU145 cancer cells, compared to DMSO as a vehicle control Once spheroids were formed, the drug responsiveness was evaluated by measuring the extent of spheroid growth in response to either DMSO as vehicle control or designated concentrations of HVS (1, 3, and 10 μM), in the presence of 33 ng/mL HGF, for a treatment period of 72 h. Spheroids were grown at 37°C and 5% CO_2_ in IncuCyte ZOOM (Essen Bioscience). Images were acquired every 4 h and software analysis was designed to identify the red object in each well. The data were expressed as fold increase in spheroids size from *T* = 0 to *T* = 72 using the “average red object area in each well” as determined by the IncuCyte software analysis. Error bars indicate the SEM of *n* = 3/dose. (−)-Oleocanthal was used as a positive control at 20 μM dose. (**C**) Representative images of the DU145 spheroids from different treatment groups at the 72 h time point are shown.

Cultures grown as 3D spheroids more closely recapitulate the *in vivo* responses to drugs. Therefore, this model was used to analyze the effects of the oleocanthal-based HVS on an implemented 3D spheroid culture system, stimulated with HGF, which might provide a better *in vivo* prediction of HVS profile. The human breast MDA-MB-231 and the prostate DU145 cancer cell lines were used to develop 3D spheroid culture models using low attachment U-bottom plates. After spheroid formation, the drug responsiveness was evaluated by measuring the extent of spheroid growth in response to either dimethyl sulfoxide (DMSO) as a vehicle control or designated concentrations of HVS (1, 3, and 10 μM) for a treatment period of 72 h. (−)-Oleocanthal was tested at 20 μM for activity comparison. Images were captured every 4 h using the Incucyte real time imaging platform (Figures S4 and S5). In case of MDA-MB-231 cells, HVS significantly reduced HGF-induced spheroid growth in a dose dependent fashion, with 80% inhibition observed at a 10 μM (Figure 6A). HVS also potently inhibited HGF-mediated DU145 spheroid growth, demonstrating superior activity (> 20-fold) over (−)-oleocanthal, which slightly reduced spheroid growth at 20 μM. HVS blocked HGF-induced DU145 spheroid growth, at concentrations as low as 1 μM, in a dose dependent fashion, with a 10 μM dose reducing spheroid growth by over 80% (Figure 6B and 6C). Thus, HVS blocked HGF-driven growth not only in two-dimensional (2D) cultures but also in 3D spheroid systems, suggesting its potential for the control of c-Met-dependent solid tumors, including breast and prostate cancers.

### Effect of HVS on c-Met phosphorylation

**Figure 7:**
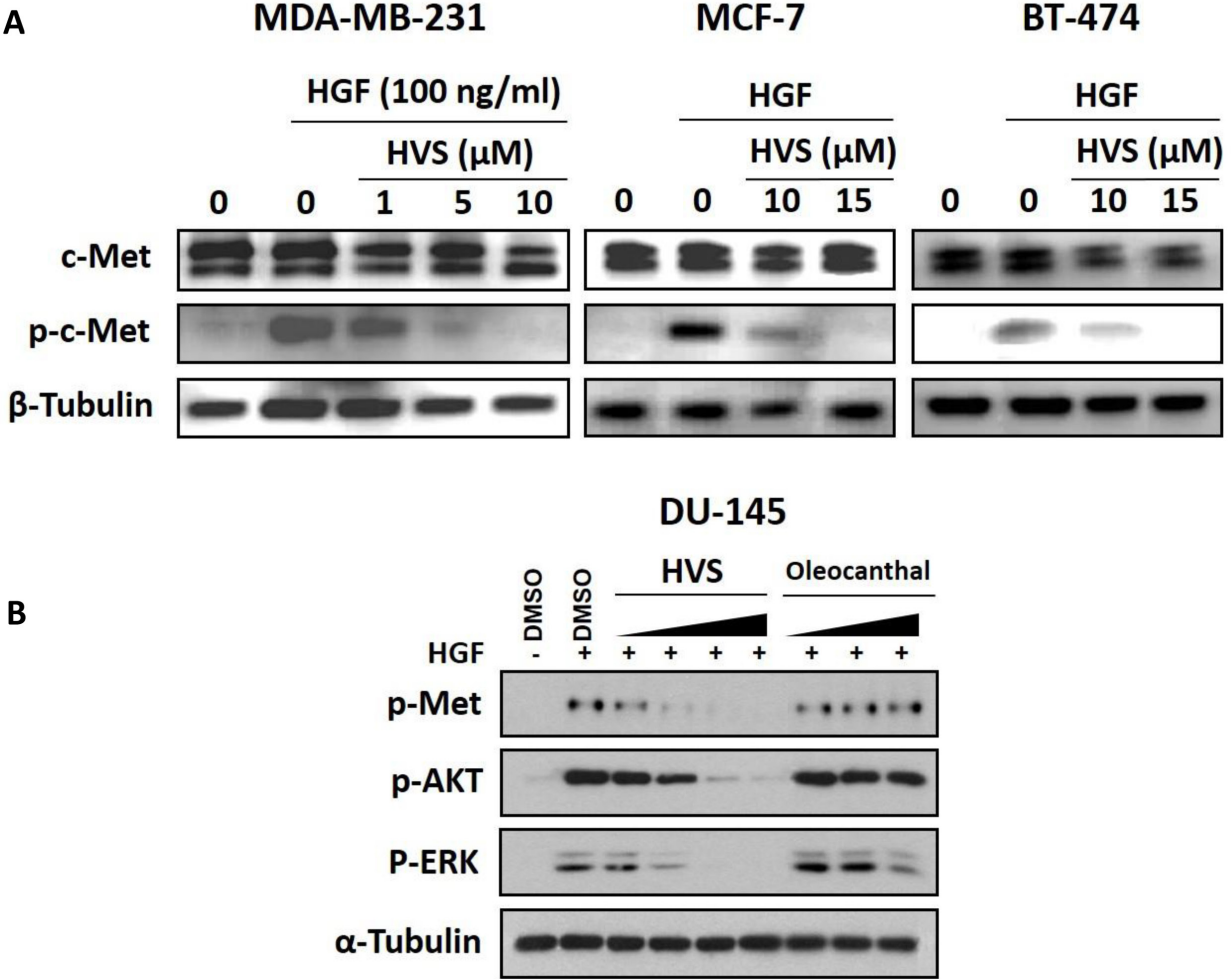
(**A**) Effect of HVS treatment on HGF-induced c-Met activation in human breast cancer cells using Western Blot analysis. Treatment with HVS caused a significant, dose-responsive inhibition of HGF-induced c-Met phosphorylation in MDA-MB-231, MCF-7, and BT-474 mammary tumor cells with no effect on total c-Met levels after treatment for 72 h, as compared to their respective vehicle-treated control groups. The visualization of β-tubulin was used as a loading control. (**B**) Effect of HVS on HGF-induced c-Met activation and its downstream mitogenic signaling in DU145 human prostate cancer cells, as compared to vehicle-treated control group, using Western Blot analysis. Cells were pre-treated for 30 minutes with either HVS at 0.1, 0.3, 1, and 5 μM or (−)-oleocanthal at 5, 10, and 20 μM doses in serum-free media. 33 ng/mL HGF was then added for 30 minutes. Whole cell lysates were collected and assayed by Western Blot. The visualization of α-tubulin was used as a loading control. Representative Western blots from each experiment are shown, *n* = 3.

In this study, MDA-MB-231, MCF-7, and BT-474 human breast cancer cells were chosen to assess the effect of HVS on HGF-induced c-Met phosphorylation using Western blot analysis, to confirm the initial biochemical assay results. Phospho-c-Met refers to the phosphorylation of the kinase domain at Y1234/1235. Cells were exposed to multiple different doses of HVS and then, the expression and phosphorylation levels of c-Met protein were determined in cell lysates (Figure 7A). Results revealed a significant and dose-responsive inhibition of HGF-induced c-Met phosphorylation after treatment with HVS for 72 h in the three used c-Met-dependent breast cancer cell lines, as compared to their respective vehicle-treated control groups, consistent with the *in vitro* kinase activity of HVS. These results also match well with the antiproliferative effects in the three cell lines, as a result of HVS's c-Met kinase inhibition (Figure 7A). Meanwhile, HVS did not affect the total c-Met levels at the same treatment doses in all breast cancer cell lines (Figure 7A).

To further assess the cellular activity of HVS against c-Met, we next measured its effect on c-Met phosphorylation in DU145 prostate cancer model, which responds well to HGF stimulation. (−)-Oleocanthal has been also tested for its ability to inhibit HGF/c-Met signaling in this cancer model. Cells were treated for 30 min with a varying concentrations of HVS (0.1, 0.3, 1, and 5 μM) or different doses of (−)-oleocanthal (5, 10, and 20 μM). Cells were then stimulated with HGF for 30 min, and the phosphorylated c-Met levels were determined in cell lysates by Western blot (Figure 7B). The results revealed a striking reduction in the activated levels of c-Met in DU145 cells after HVS treatment. HVS potently inhibited HGF-induced c-Met phosphorylation, in a dose-dependent manner, with sub-μM concentrations effectiveness, compared to the vehicle-treated control group (Figure 7B). Surprisingly, (−)-oleocanthal did not show a significant inhibitory effect on c-Met activation in this prostate cancer model, corroborating the results of the scattering assay and suggesting that (−)-oleocanthal would interrupt c-Met signaling in a cell type-specific manner (Figure 7B).

### Effect of HVS on downstream c-Met signaling pathways

**Figure 8:**
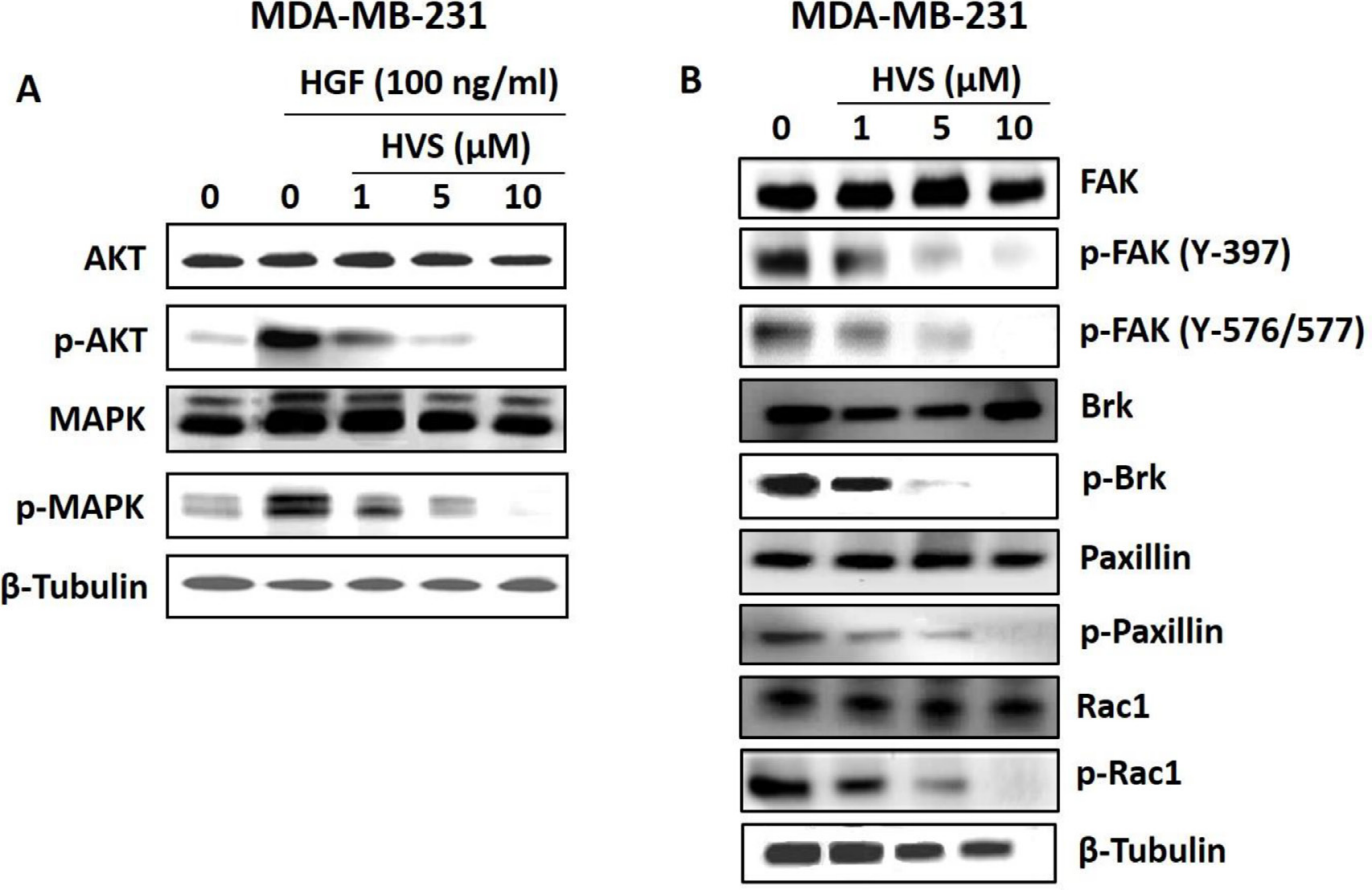
Effects of HVS treatment on c-Met downstream mitogenic and FAK/Brk/paxillin/Rac1 signaling pathways in human MDA-MB-231 breast cancer cells after a treatment period of 72 h (**A**) Western Blot analysis showing HVS treatment effects on c-Met downstream mitogenic signaling molecules, Akt and MAPK, using different treatment doses, compared to the vehicle-treated control group. (**B**) Western Blot analysis showing HVS treatment effects on FAK/ Brk/ Paxillin/Rac1 signaling pathway using different treatment doses, compared to the vehicle-treated control group. The visualization of β-tubulin was used as a loading control. Representative Western blots from each experiment are shown, *n* = 3.

The identification of HVS as a promising c-Met inhibitor offered unique opportunities to investigate the pharmacologic consequences of c-Met inhibition. Upon HGF binding, c-Met dimerizes, transphosphorylates, and activates downstream signaling through several pathways with diverse cellular functions that are important in cancer progression, including the Ras-related C3 botulinum toxin substrate 1 (Rac1)/ cell division control protein 42 homolog (Cdc42) pathway, the phosphoinositide-3 kinase (PI_3_K)/protein kinase B (Akt) pathway, breast tumor kinase (Brk) and phospholipase C-γ pathways as well as the extracellular signal regulated kinase (ERK)/mitogen-activated protein kinase (MAPK) cascade [2, 3, 8]. Together, these pathways regulate cellular proliferation, migration, invasion and tubulogenesis [2]. In particular, Akt, MAPK and Brk signaling molecules are necessary not only for the c-Met-mediated regulation of cell motility, adhesion, and invasion, but also for the control of cell survival and mitogenesis [6, 7, 29]. In this study, the human breast MDA-MB-231 and prostate DU145 cancer cells were selected to evaluate the effects of HVS treatment on c-Met-dependent signaling pathways using Western blot analysis (Figures 7B and 8).

### Effect of HVS on proliferation and survival markers

The effects of HVS on Akt and ERK/MAPK signaling were studied by Western blot analysis (Figures 7B and 8A). MDA-MB-231 breast cancer cells were exposed to different doses of HVS for 72 h. HVS treatment caused a dose-dependent inhibition of HGF-induced phosphorylation of Akt and ERK/MAPK, which are important c-Met signaling downstream molecules essential for cell survival and proliferation, respectively. On the other hand, HVS treatment did not affect the total levels of Akt and MAPK at the same treatment doses in MDA-MB-231 cells (Figure 8A). The phosphorylation levels of Akt and ERK were determined in DU145 prostate cancer cell lysates after treating cells with various concentrations of HVS or *S*-oleocanthal for 30 min followed by HGF stimulation before cell lysis. HVS treatment potently inhibited HGF-induced phosphorylation of Akt and ERK, in a dose-dependent manner, as compared to the vehicle-treated control group in DU145 cells (Figure 7B). *S*-Oleocanthal was nearly without any significant effect on Akt phosphorylation while it appeared to have a little effect on activated ERK levels only at relatively high treatment dose (20 μM) in this prostate cancer model, compared to vehicle-treated cells (Figure 7B).

### Effect of HVS on motility markers

To study the effects of HVS on focal adhesion kinase (FAK)/Brk/paxillin/Rac1 pathways, Western blot analysis was performed (Figure 8B). MDA-MB-231 cells were exposed to different doses of HVS for 72 h. Consistent with c-Met phosphorylation inhibition, the results of this study demonstrated a dose-dependent suppression of FAK, Brk, paxillin, and Rac1 phosphorylation after HVS treatment, compared to the vehicle-treated control group (Figure 8B). Alternatively, HVS had little or no effects on the total levels of FAK, Brk, paxillin, and Rac1 in treated cells (Figure 8B). Collectively, these data indicated that the oleocanthal-based HVS is a robust inhibitor of the HGF/c-Met signaling axis and its important downstream pathways mediating proliferation, survival, and motility in different cancer models, with superior activity over *S*-oleocanthal, especially in prostate cancer model, consistent with the scattering and spheroid growth assay results.

### Selectivity assessment of HVS

The remarkable potency of HVS for inhibition of ATP binding and c-Met autophosphorylation prompted us to investigate whether this potency was specifically against c-Met. Accordingly, HVS was evaluated against a cross-section of tyrosine kinases (TKs), which are known to be structurally related and oncogenically relevant to c-Met, in order to understand its selectivity profile (Table 2). A panel of 15 TKs was selected for biochemical analysis through the SelectScreen Kinase Profiling Service (Invitrogen, CA). In this assay, the results are expressed as % inhibition at a 10 μM dose (Table 2). Significant inhibition (> 50%) at 10 μM of HVS was observed only for abelson murine leukemia viral oncogene homolog 1 (ABL1) and c-Met, with greatest relative inhibition for ABL1 (Table 2). In contrast to its high potency against c-Met and ABL1, HVS barely inhibited the other tested TKs activity, including the c-Met family member RON and the highly homologous, phylogenetically related kinase TYRO3 (3~4-fold selectivity difference versus c-Met, Table 2). HVS was determined to be more than 60-fold selective for c-Met versus insulin-like growth factor receptor 1 (IGF1R) and v-kit Hardy–Zuckerman 4 feline sarcoma viral oncogene homolog (KIT), whereas all other kinases evaluated in the panel, except ABL1, were found to be 3–16 fold less sensitive to HVS compared with c-Met. Due to the expression of ABL1 in all cancer cell lines investigated in this study, the relevance of this kinase in mediating the pharmacologic activity of HVS cannot be discounted [30, 31]. These results revealed that HVS demonstrated exquisite selectivity by only targeting two kinases, c-Met and ABL1, at pharmacologically relevant concentration, and suggested that HVS might be a promising c-Met/ABL1 dual kinase inhibitory hit.

**Table 2:** Initial TK-selectivity profile of HVS

Kinase	Mean inhibition at 10 μM (%)
ABL1	69
ALK	21
EGFR (ERbB1)	7
HER2 (ERbB2)	4
HER4 (ERbB4)	7
FGFR1	20
FLT1 (VEGFR1)	9
IGF1R	−5
KDR (VEGFR2)	20
KIT	−3
MET (c-Met)	62
MST1R (RON)	15
PDGFRβ (PDGFR beta)	14
ROS1	24
TYRO3 (RSE)	21

ErbB, erythroblastic leukemia viral oncogene homolog; FGFR1, fibroblast growth factor receptor 1; FLT1, fms-like tyrosine kinase; KDR, kinase insert domain receptor; RON, recepteur d'origine Nantais; MST1R, macrophage stimulating 1 receptor; PDGFR, platelet-derived growth factor receptor; TYRO3, tyrosine protein kinase; RSE, receptor sectatoris.

**Figure 9:**
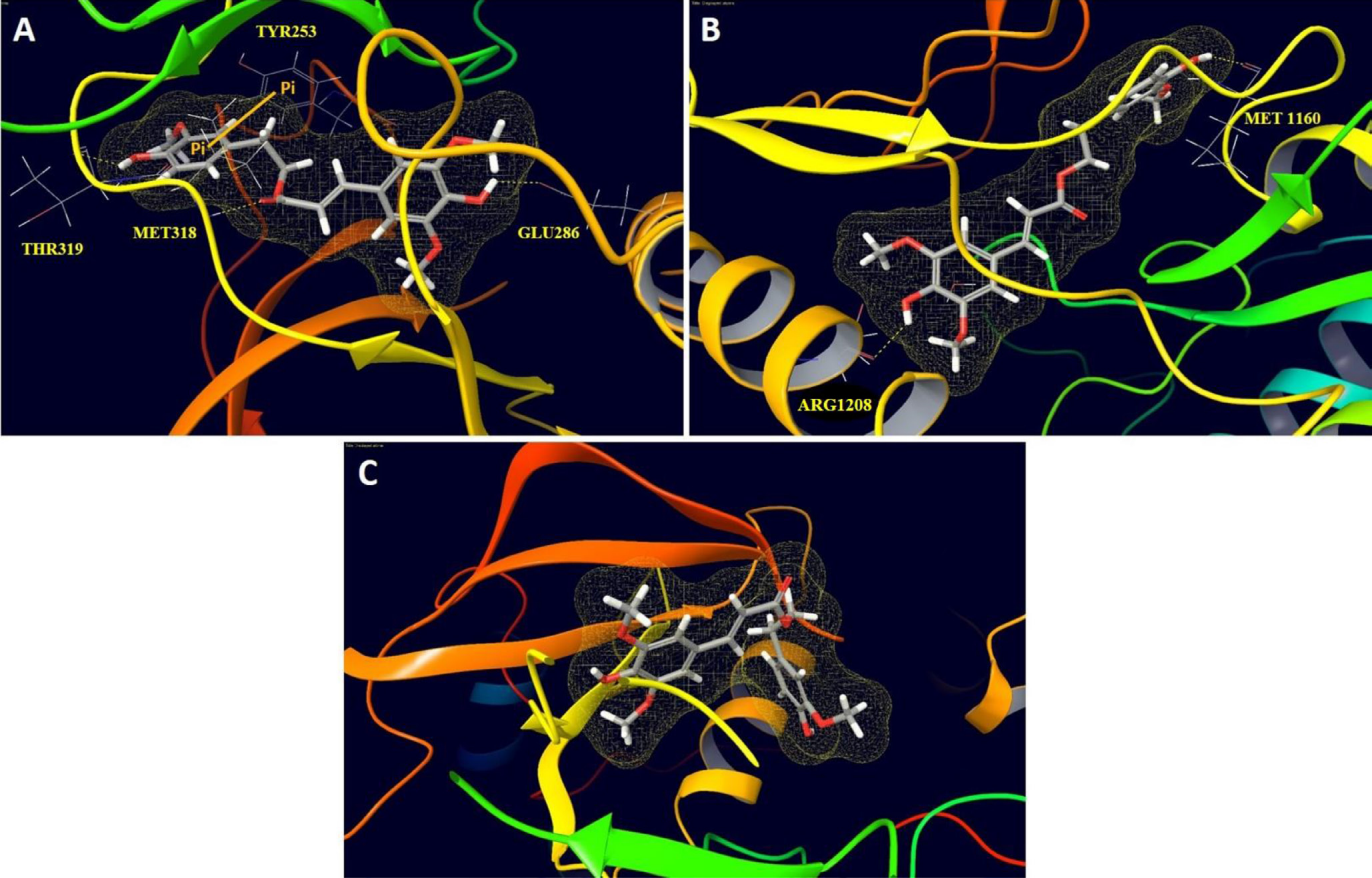
Peptide backbone structural models of the ATP binding sites of (A) ABL1, (B) c-Met, and (C) IGFR1 with HVS docked in the ATP binding site The solvent-excluded volume in the binding site, as calculated by the program SiteMap [33], is outlined in yellow mesh. This figure was created using Maestro 9.3 panel interface (Maestro, version 9.3, 2012, Schrödinger).

To identify the structural basis for HVS's ABL1 affinity, additional docking simulation studies have been carried out using Glide extra-precision (XP) (Schrödinger 2014). Interactions of HVS with TKs for which it demonstrated the highest and lowest affinities, including ABL1 (69% inhibition), c-Met (62% inhibition) and IGF1R (no observed inhibition) were studied. The crystal structures of ABL1 bound to the inhibitor ponatinib (PDB code: 3OXZ) and of IGF1R bound to a non-hydrolysable ATP analog (PDB code: 1JQH), in addition to the co-crystal structure of 8-fluorotriazolopyridine bound to c-Met (PDB code: 4XYF) for comparison were chosen. Both docking scoring functions available in Glide, ChemScore and GlideScore, were used since the combination of multiple scoring functions provides better prediction and correlation between docking scores and ligand affinity [32]. These scoring functions proved good agreement between docking score/fitness results and experimental affinity data obtained from the selectivity profiling screen for the three kinases (Table 3). HVS received the lowest score of −1.0 when docked into the crystal structure of IGF1R, while it had a higher modeling score of −8.1 upon docking into the crystal structure of c-Met and the best score of −11.4 upon binding to ABL1 crystal structure, consistent with selectivity profiling screen results (Tables 2 and 3). The binding site of each kinase was evaluated by calculating various properties using the program SiteMap (SiteMap 2.9, Schrödinger 2014) to better understand functional groups contributed to binding differences. SiteMap generates information on the characters of binding sites using novel analysis facilities and provides information to Maestro for sites visualization [33]. The most important property generated by SiteMap is an overall SiteScore, which effectively identify known binding sites in co-crystallized complexes. A score of > 1 suggests a site of particular promise for a particular small-molecule, whereas a SiteScore of 0.8 has been found to accurately distinguish between drug-binding and non-drug-binding sites [33]. Other properties characterize the binding site in terms of its size and volume using a grid of site points, which fill the binding site cavity with a set of solvent-excluded probe spheres approximating its volume [33]. The number of site points that make up the site is a measure of its size. The size and volume of the site are often good indicators of the preferred binding site for the tested small-molecule [33]. Calculation of various binding site properties and detailed examination of HVS binding pose in each of the three kinases revealed key structural features which contributed to differences in binding affinity toward the three kinases (Table 3, Figure 9). HVS fits very well in the binding site of ABL1 kinase, displaying proper complementarity to the receptor and forming several critical interactions predicted to stabilize the kinase domain in its inactive conformation and thus, prevents its ATP activation (Figure 9A). Docking of HVS into ABL1 kinase revealed a HB interaction between its C-6′ phenolic hydroxyl and the backbone carbonyl of Thr319 in the hinge region. Additionally, the ester carbonyl contributed a critical HB interaction with the backbone of Met318 at the hinge region while the aromatic C-7 phenolic hydroxyl group formed a HB with the side chain carboxylate of Glu286. Furthermore, the terminal aromatic ring A of HVS is oriented along the surface edge of the binding site, making a strong π-π stacking interaction with Tyr253, which has an important role in stabilizing the inactive form of the kinase (Figure 9A). HVS binds to ABL1 domain via four important interactions whereas it exhibited only three interactions upon binding to wild-type c-Met and its oncogenic variant, which might explain, at least in part, the improved activity to ABL1 compared to c-Met (Figure 9A and 9B). SiteMap ABL1 and c-Met binding sites calculations and volume visualizations suggest that HVS is buried more deeply in the binding site of ABL1 than observed for c-Met (Figure 9 and Table 3). The hydrophobic pocket where HVS fits is deeper and larger in ABL1 than in c-Met, as illustrated by comparing the properties of both sites which showed that the volume and size of ABL1 binding site are significantly larger compared to c-Met (Table 3). The larger and deeper pocket of ABL1 allowed an exceptional fitting of the HVS, facilitating several favorable interactions with Val256, Ala269, Leu370, Ala380, Phe317, Val299, and Phe382 which in turn, stabilized the bioactive U-shaped conformation and conferred a substantial binding affinity towards ABL1 (Figure 9A). These observations can explain the higher docking score and improved activity of HVS against ABL1 compared to c-Met. However, the c-Met pocket was still able to accommodate HVS with a small conformational shift due to the plasticity of this kinase in molding the activation and nucleotide-binding loops to the shape of the ligand in the binding site. In contrast, the aforementioned hydrophobic pocket was very shallow or essentially nonexistent in IGF1R as confirmed by the calculated volume of the site, and thus, HVS has been imperfectly docked upside down with its main scaffold outside the binding site, unable to demonstrate critical interactions with the protein, consistent with the low observed activity and docking score for this kinase (Figure 9C, Table 3). Additionally, the calculated SiteScore for IGF1R binding site was significantly smaller than those of c-Met and ABL1, indicating its lower potential to accommodate drug-like molecules with HVS size (Table 3). Interestingly, the HVS C-5′ methoxyl group was solvent exposed, upon binding to ABL1 (Figure 9A), and therefore should not have a significant impact on ABL1 inhibition unlike c-Met, where the same methoxyl group nicely fits into a hydrophobic sub-pocket created by Ile1084, Phe1089, and Val1092 (Figure 3A) and thus, caused remarkable c-Met inhibition improvement, compared to compounds lacking this functionality, in c-Met biochemical assays [34]. This observation suggested that the selectivity of HVS analogs could be tuned to favor either ABL1 or c-Met by adjusting the phenyl ring A C-5′ substituents size and nature. Such modifications could presumably affect binding affinity to the clinically relevant c-Met mutant V1092I as well, in which the size of the hydrophobic sub-pocket is likely to be reduced. Better understanding of the selectivity structural determinants is the first step toward the rational design of more potent and selective c-Met inhibitors structurally related to HVS.

**Table 3:** Binding site evaluation using SiteMap and modeling scores upon HVS binding

SiteMap Property	Kinase		
	IGF1R	MET	ABL1
SiteScore	0.78	1.125	1.137
Binding pocket size (number of site points)	85.0	148.0	186.0
Binding pocket volume (mm^3^)	252.12	297.04	409.12
Modeling ChemScore	−1.0	−8.1	−11.4
Modeling GlideScore	+1.0	−7.2	−10.9

### *In vivo* antitumor activity of HVS

**Figure 10:**
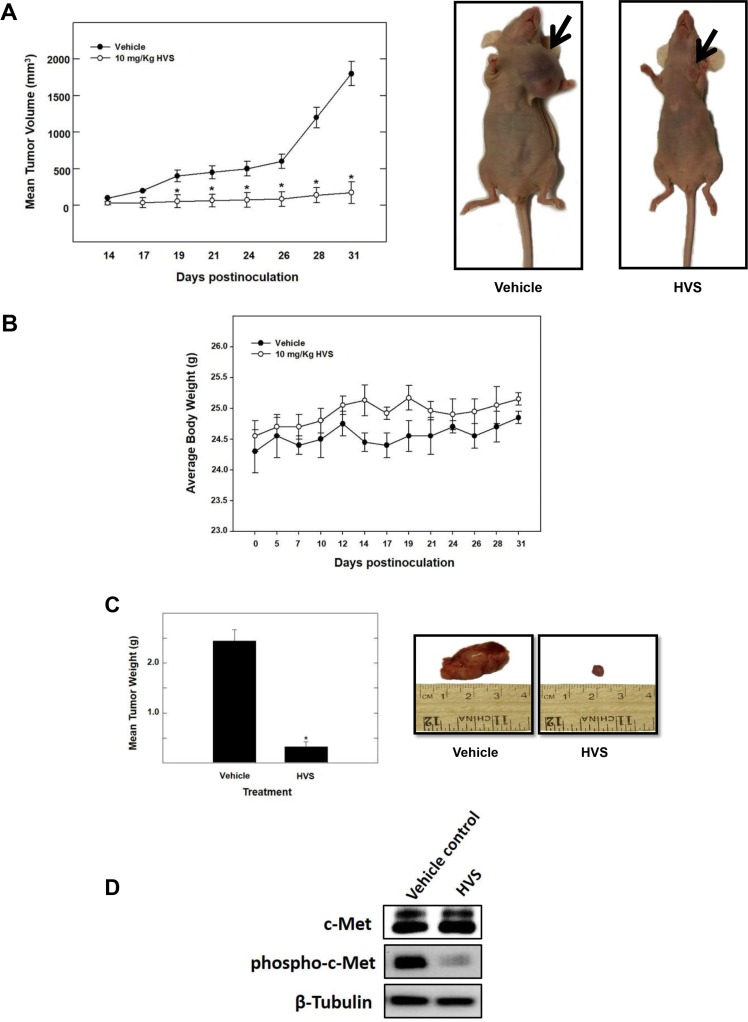

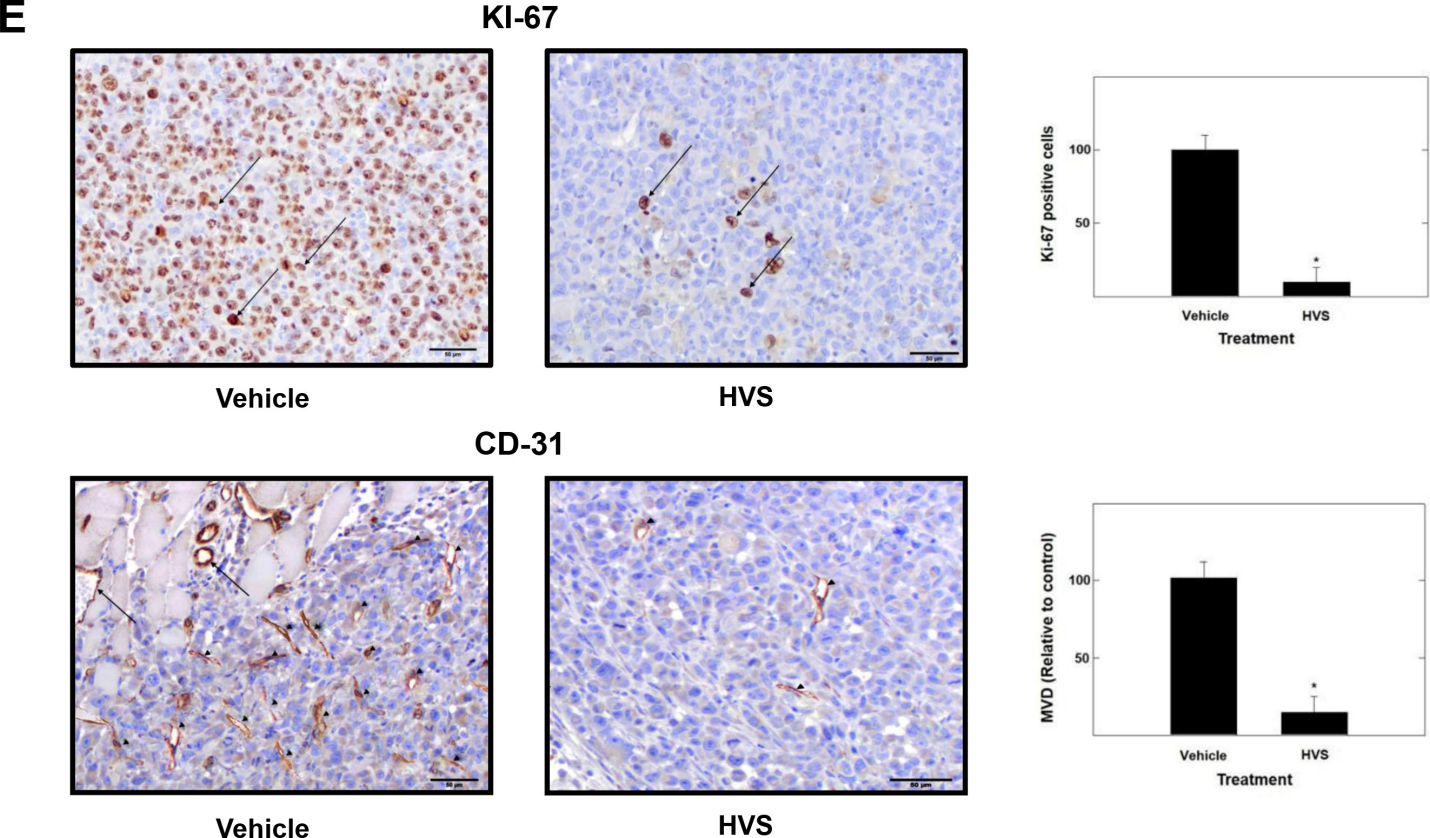
The effect of HVS treatment on tumor growth in the human breast cancer xenograft model Athymic nude mice with subcutaneous orthotopic MDA-MB-231/GFP human breast cancer cells were intraperitoneally injected with 10 mg/kg of HVS or the vehicle control. Treatment regimens were administered 3×/week, starting 5 days postinoculation. (**A**) *Left panel*; tumor volume was evaluated periodically during treatment at the indicated days postinoculation. Tumor volume (V) was calculated by *V* = (L × W^2^)/2, where L was the length and W was the width of tumors. Points, the mean of tumor volume in mm^3^ of several tumors (*n* = 5) during the course of the treatment period; bars ± SEM. *P < 0.05 as compared to vehicle-treated control. *Right panel*; shown are two mice harboring human breast cancer. The mouse on the right shows the suppression of tumor growth with HVS treatment (10 mg/kg/day) compared to the vehicle-treated control mouse on the left. (**B**) No significant change in body weight was observed among treated animals, indicating the safety of HVS treatment. Error bars indicate SEM for *n* = 5. (**C**) *Left panel*; vertical bars indicate mean tumor weight at the end of the experiment. *P < 0.05 as compared to the vehicle-treated control. Error bars indicate SEM for *n* = 5. *Right panel*; shows photomicrographs of primary breast tumors from mice with vehicle-treated cancer (left), and cancer treated with HVS at 10 mg/kg/day (right). (**D**) Protein expression of total and phosphorylated levels of c-Met in vehicle-treated or HVS-treated (10 mg/kg/day) breast tumors detected by Western blot. (**E**) *Left panel*; immunostaining of sections obtained from vehicle-treated or HVS-treated (10 mg/kg/day) mice against Ki-67 (mitosis marker) and CD31 (endothelial marker) antibodies. *Right panel*; shows quantification of Ki-67 positive cells and microvessel density (MVD). Ki-67 positive cells in breast cancer tissues were examined in 5 areas at a magnification of ×200. MVD of breast tumor tissue sections was evaluated. Any CD31+ stained endothelial cell or endothelial cell cluster was counted as one microvessel. The mean microvessel count of the five most vascular areas was taken as the MVD, which was expressed as the absolute number of microvessels per 1.485 mm^2^ (×200 field). Vertical bars indicate the average of 5 readings ± SEM, *P < 0.05 as compared with vehicle-treated controls.

The encouraging *in vitro* results of HVS in inhibiting c-Met mediated neoplastic phenotypes warranted further *in vivo* studies to evaluate its antitumor efficacy including pharmacodynamics (PD), immunohistochemical analysis and tumor growth inhibition (TGI) in a mouse model. Efficacy studies were carried out in a xenograft orthotopic athymic nude mouse model using MDA-MB-231/GFP human breast cancer cells, a representative of cancer in which c-Met is dysregulated. Dosing (10 mg kg^−1^ i.p., 3 times per week) started 5 days post-inoculation and continued for 4 weeks. The mice were monitored by measuring body weight, and clinical observation every other day. Tumor progression was followed by direct measurement of tumor volume starting 14 days after the orthotopic tumor inoculation. Breast tumor growth was compared between non-treated animals and those receiving HVS treatment (Figure 10A). Following HVS administration cycle, the results demonstrated a significant reduction in both tumor volume and tumor weight as compared to the vehicle-treated control group (Figure 10A and 10C). In this study, 10 mg/kg of HVS suppressed the MDA-MB-231 tumor growth by 92% on the final day of study, compared to the vehicle-treated control group, without adversely affecting the treated mice's normal body weight or their gross phenotype, indicating that HVS can demonstrate a good efficacy against breast cancer in clinically relevant orthotopic mouse model (Figure 10A–10C).

### c-Met pharmacodynamics study

*In vivo* target modulation studies were carried out in nude mice bearing MDA-MB-231 tumor xenografts by determining the effect of HVS treatment on c-Met phosphorylation. Pharmacodynamically, HVS at 10 mg/kg, 3×/week, i.p., significantly inhibited the phosphorylation of c-Met kinase in human breast MDA-MB-231 xenograft tumors when compared to the vehicle treated control group, as shown by Western blot analysis of the isolated tumor tissues, without any change in total c-Met levels (Figure 10D). These results revealed that the *in vitro* c-Met inhibitory potency of HVS could be successfully translated into *in vivo* activity.

### Immunohistochemistry

Consistent with marked suppression of tumor growth and the observed pharmacodynamic inhibition of the c-Met phosphorylation, immunohistochemical analysis of the tumor specimens revealed that HVS treatment suppressed both mitosis and new vessel formation, as evidenced by the significant reduction of the expression of their markers, Ki-67 and CD-31, respectively, compared to the vehicle-treated group (Figure 10E). HVS treatment significantly decreased the tumor microvessel density (MVD), calculated by new vessel formation using CD-31 staining (Figure 10E).

## DISCUSSION

The accumulation of information implicating c-Met as a major regulator of tumor progression and the prevalence of HGF/c-Met activation in human malignancies continue to drive the search for more effective c-Met inhibitors, since its first discovery in 1984. One way to effectively block c-Met signaling is by inhibiting its catalytic activity with small-molecule inhibitors. Inspired by the chemical structure of *S*-oleocanthal (Figure 1), a natural olive secoiridoid with documented activity against c-Met-dependent malignancies, the data presented herein identify the oleocanthal-based HVS as a novel small-molecule c-Met inhibitor lead in multiple cancer models. HVS stood out as a potent c-Met inhibitor resulting in a marked inhibition of the phosphorylation of a peptide substrate recognized by recombinant human wild-type c-Met kinase and its oncogenic variant M1250T, with IC_50_ values of 1 and 0.9 μM, respectively (Figure 2). HVS showed superior activity over *S*-oleocanthal in these cell-free assays. One mechanism by which c-Met deregulation leads to cancer is through gain-of-function mutations which is often correlated with poor clinical outcomes [13]. Various activating point mutations in the c-Met proto-oncogene have been implicated as the cause of different types of cancers [35]. The presence of striking homologies between Met proto-oncogene mutations and mutations in other RTKs has already been documented [36, 37]. Three of these mutations (D1228N, D1228H, and M1250T) are located in codons homologous to those mutated in the tyrosine kinase receptors Kit and Ret, suggesting that these amino acids are structurally conserved hotspots for mutations. Several more recent literature examples proved that c-Met inhibitors show differential ability to modulate the activities of wild and mutant-type kinases, which can either induce primary drug resistance in case of pre-existing mutations or acquired resistance due to chronic treatment [14, 38]. Therefore, discovery of additional novel small-molecule c-Met inhibitors capable of targeting these mutations is still needed to overcome the predicted resistance and offer therapeutic benefits for affected patients. The ability of HVS to inhibit the oncogenic human c-Met mutant M1250T with almost equal potency compared with the wild-type receptor in cell-free assays, supported by *in silico* docking studies (Figures 2B and 3, respectively), provides a stepping stone to: broaden the oleocanthal-based esters therapeutic scope as anticancer agent; cover clinical populations with higher expression levels of this mutant variant; and offer efficacy insights for use to control aggressive tumor phenotypes expressing mutant c-Met constructs. However, additional studies will be needed to determine whether this mutant inhibition in cell-free system can be recapitulated in cells engineered to overexpress mutant c-Met constructs such as those found in hereditary papillary renal cell carcinoma patients. c-Met inhibitors represented by SU11274 exhibit uncompromised inhibitory activity against this c-Met mutant but they have been limited to *in vitro* or brief *in vivo* studies and were not viable clinical candidates due to poor pharmaceutical properties and oral bioavailability [39]. HVS, which is structurally distinct from SU11274, represents a new scaffold with unique pharmacophores with this desirable biological property. Together, HVS is a promising c-Met inhibitory hit appropriate for further molecular level validation to determine whether its activity against mutant c-Met in cell-free assays could be recapitulated *in vitro* and *in vivo*.

Results demonstrated the ability of HVS treatment to significantly inhibit HGF-induced proliferation across a broad spectrum of c-Met expressing human breast cancer cell lines in a dose-responsive manner (Figure 4A). Meanwhile, HVS treatment had little or no discernable effects on the growth and/or the viability of the non-tumorigenic human mammary epithelial cells at a concentration which was numerous-fold higher than the growth inhibitory concentrations against the neoplastic breast cancer cells (Figure 4D). The human triple negative breast cancer (TNBC) MDA-MB-231 and MDA-MB-468 cells, with higher levels of c-Met expression, are the most sensitive to the antiproliferative effects of HVS compared to other breast cancer cells with lower c-Met levels. TNBC is characterized by the lack of the expression of ER, progesterone, and HER-2 receptors [40, 41]. It accounts for ~17% of all breast cancers, representing an aggressive clinical phenotype and is generally associated with poor prognosis. Thus, chemotherapy remains the only systemic treatment option available for TNBC patients [40, 41]. Accordingly, there is a dire need to develop new treatments for this aggressive subtype, which currently lack targeted therapy. The effect of HVS treatment on MDA-MB-231 cells’ viability and LDH release was quantified. LDH is a soluble cytosolic enzyme, which is released into the surrounding culture medium upon cell damage or lysis during apoptosis and necrosis. Therefore, the detection of LDH in the culture medium can be used as an indicator of cell membrane integrity and serves as a marker for cytotoxicity. HVS induced minimal LDH release at 30 and 60 μM concentrations in MDA-MB-231 cells (Figure 4C). These concentrations represent several-fold above HVS's antiproliferative IC_50_, suggesting lack of mitochondrial mechanism of cytotoxicity and hence HVS induces cell growth arrest rather than cellular death in mammary cancer cells at achievable anticancer doses.

The inhibition of mammary cancer cell growth was associated with the ability of HVS to disrupt c-Met receptor phosphorylation in response to its natural ligand HGF in MDA-MB-231, MCF-7, and BT474 breast cancer cells (Figure 7A) and inhibit the downstream c-Met effectors in culture (Figure 8). Interestingly, the concentrations of HVS required to induce 50% inhibition of cancer cell growth were significantly greater when breast cancer cells maintained in HGF-free media as compared to those maintained in media supplied with HGF as a mitogen (Figure S3). This finding indicated that HVS treatment is only dependent on the presence of HGF, confirming its proposed molecular mechanism as a direct inhibitor of the HGF/c-Met signaling pathway. Consistently, T-47D breast cancer cells without detectable c-Met protein expression were markedly less sensitive (> 10-fold) to the antiproliferative effects of HVS compared to those expressing c-Met, confirming the differential sensitivity to c-Met-dependent human breast cancer cells. The biological functions of the HGF/c-Met signaling are mediated through a variety of downstream effectors such as Akt and MAPK. Exposure to growth inhibitory concentrations of HVS markedly inhibited HGF-induced phosphorylation and activation of Akt and MAPK in MDA-MB-231 mammary cancer cells (Figure 8A). It is well-established that HGF/c-Met signaling for mitogenesis and growth occurs through the MAPK signaling pathway [42, 43]. In addition, activation of c-Met prevents apoptosis and maintains cancer cell survival through activation of PI_3_K and subsequent Akt-Nuclear factor κB (NFκB) activation [44, 45]. Accordingly, HVS treatment effectively blocked mitogenesis and proliferation through suppression of HGF-induced c-Met phosphorylation and subsequent activation of its downstream mitogenic signaling pathways (Figures 7A and 8A).

Spheroids are one of the best characterized models for 3D cell culture where clusters of cell colonies will undergo self-assembly to form viable 3D tumor-like structures [46]. Cultures grown as 3D spheroids more accurately mimic the natural tumor microenvironment compared to 2D monolayer cultures [47]. Consistent with the inhibition of proliferation of c-Met harboring breast cancer cell lines, HVS dose-dependently inhibited the c-Met-mediated invasive MDA-MB-231 spheroid growth in 3D cultures (Figure 6A). HVS was additionally tested in 3D spheroid assays against DU145 prostate cancer cells with aberrant c-Met activity. Interestingly, HVS blocked the HGF-induced DU145 spheroid growth with superior activity over its parent *S*-oleocanthal (Figure 6B and 6C). Furthermore, HVS effectively inhibited c-Met activation and its key downstream Akt and ERK signaling cascades in DU145 cells (Figure 7B), suggesting its ability to interrupt c-Met signaling regardless of the mechanistic complexity of c-Met activation across different cellular contexts. These results provide compelling evidence that blocking the HGF/c-Met axis will enable HVS to exert its antitumor effects across a broad spectrum of c-Met-driven tumors.

HGF-induced scattering and motility is a tightly controlled process mediated by multiple effectors including Rac, Rho-associated, coiled-coil-containing protein kinase (Rock), and Brk [8, 43]. Brk is a potent inducer of migration and invasion [29, 48]. This function is mediated in part by its phosphorylation of paxillin, which leads to the activation of Rac1 via the adaptor protein, creatine kinase (CrkII) [29]. FAK is a non-receptor protein tyrosine kinase which has been implicated in controlling integrins and growth factors receptor-mediated biological processes, including cell spreading, motility, migration, differentiation, angiogenesis, and survival [49]. FAK regulates cell migration via its autophosphorylation at Tyr397 (Y397), which subsequently phosphorylates other sites of FAK, including its kinase domain, thereby triggering the activation of cell migration signaling pathways [49]. FAK also functions as a scaffolding protein associating with P130 Crk-associated substrate (P130Cas), paxillin, and other adaptors to promote cell migration and adhesion [50]. The cross-talk between Brk and FAK promotes breast tumor metastasis [51]. In addition to Akt and MAPK signaling, both Brk and FAK control the ability of malignant cells to become invasive by activating paxillin and Rac1 [29, 50]. The results of this study showed that HVS treatment caused a marked dose-dependent inhibitory effect on HGF-induced migration and invasion of MDA-MB-231 breast cancer cells (Figure 5A and 5B). The antimigratory and anti-invasive activities of HVS appeared to be mediated, at least in part, through suppression of FAK, Brk, paxillin, and Rac1 phosphorylation in response to HGF stimulation in MDA-MB-231 cells (Figure 8B). In addition, HVS substantially impaired c-Met-mediated scattering phenotype of DU145 prostate cancer cells in a dose-responsive fashion, while similar treatment doses did not affect EGF-mediated cell scattering, confirming a direct inhibition of the HGF/c-Met signaling pathway (Figure 5C). Overall, these data support the hypothesis that c-Met signaling is a primary therapeutic target for HVS *in vitro*.

Most clinically evaluated c-Met kinase inhibitors are not very selective for c-Met and exhibit activities against multiple kinases, often induce undesired off-target toxicity. Although the strategy of developing tyrosine kinase inhibitors with unrivalled specificity towards c-Met offers theoretical advantages in limiting the confounding issue of off-target toxicity and could specifically achieve the therapeutic potential of c-Met inhibition alone, it is possible that many such compounds will have limited efficacy as single-agent therapies, due in part to the development of drug-resistant mutations in tumor cell lines. Thus, engineering a TK inhibitor with selectivity for a small group of known, disease-critical kinases may offer the advantages of broad action without the development cost of singularly targeted agents or the potential toxicity of broad-acting agents with poorly defined selectivity. In addition to c-Met, HVS significantly inhibited ABL1, in a panel of sequence-related kinases screened in this report, which could prove to be a very desirable feature of a small-molecule c-Met antagonist. ABL1 is a member of the mammalian Abelson family of non-receptor TKs, which also includes ABL2 [52]. ABL1 was first identified as an oncogene required for the development of human leukemia initiated by retroviruses or chromosome translocations [52]. ABL1 transduces diverse extracellular signals to protein networks that control proliferation, survival, migration, and invasion [52]. Recent reports have uncovered roles for ABL kinases in solid tumors [30, 31, 52]. Enhanced expression and activation of ABL1 kinase have been implicated in the progression of a wide variety of solid tumor types where c-Met activation also occurs, including breast and prostate cancers [31, 52]. In solid tumor cells, ABL1 activation has often been linked to upstream stimulation by hyperactive oncogenic RTKs and chemokine receptors [31, 53]. Activation of ABL1 kinase in NSCLC and breast cancer malignancies has been shown to occur downstream of the EGFR, human epidermal growth factor receptor 2 (HER2), and IGFR, as a late occurring event that contributes to the aggressive growth and metastasis of these solid tumors [31, 52, 53]. Accordingly, ABL1 kinase can be considered as a point of convergence of the EGFR, HER2, and IGFR pathways and therefore it is an ideal target versus trying to inhibit three separate pathways [54]. ABL1 was also proposed to interconnect oncogenic Met and p53 pathways in cancer cells [55]. Based on these data, HVS would be capable of not only targeting its primary therapeutic target, c-Met, but also of interrupting multiple important pathways in solid tumor signaling, via blocking ABL1 activation, which adds a significant advantage over currently approved c-Met inhibitors in controlling aggressive phenotypes of solid tumors. Additionally, inhibition of ABL1 and c-Met sensitizes solid tumor cells to conventional agents and overcomes drug resistance developed to anticancer therapies already in clinical use, including EGFR and BRAF kinase inhibitors [10, 30, 31]. Therefore, we foresee that a dual c-Met/ABL1 inhibitor, such as HVS, could not only be initially effective in slowing tumor progression but also could prevent drug-resistant tumor growth and might be more effective if used in combination with chemotherapeutic agents for aggressive solid tumor phenotypes. Although dual inhibition of c-Met and ABL1 sounds like attractive strategy for cancer therapy, there have not been known c-Met/ABL1 dual inhibitors, rendering HVS one of the novel dual TK inhibitors known to date. Inhibitors with combined activity against c-Met and ABL1, such as HVS, could be a novel and logical target combination for treating a variety of human cancers. Meanwhile, the present study identified the structural determinants for selectivity and provided future guidance to chemically improve affinity and selectivity profiles of oleocanthal-based esters (Figure 9).

HVS treatment showed a robust antitumor efficacy that was correlated with the inhibition of c-Met-mediated signaling in an orthotopic model of MDA-MB-231 breast cancer xenograft in female athymic nude mice (Figure 10). HVS administration in experimental animals resulted in a clear suppression of tumor growth as compared to the vehicle-treated control animals. These results were associated with a significant reduction in cancer cell proliferation and mitosis markers, as shown by Ki-67 immunostaining study. Ki-67 is a nuclear protein uniquely expressed in proliferating cells, with peak concentrations in the G2 and M phases of the cell cycle and thus, it can be considered as intratumoral proliferation index [56]. Angiogenesis, or neovascularization, is known to play an important role in tumor growth and neoplastic progression leading to metastasis [57]. CD-31 is a validated endothelial cell marker shown to be a sensitive and specific indicator of endothelial differentiation [58]. HVS caused a marked reduction in microvessel formation in the tumors as indicated by reduction in the expression of the angiogenesis marker CD-31 in treated animals, suggesting a potential antiangiogenic mechanism for this compound *in vivo*. In agreement with cellular data, HVS-treated mice revealed a marked reduction in the intratumoral c-Met phosphorylation in comparison with the control mice, suggesting a good pharmacodynamics profile. Consistent with the *in vitro* lack of toxicity against the non-tumorigenic MCF10A mammary epithelial cells, nude mice showed good tolerance to the HVS treatment. Intraperitoneal administration of 10 mg/kg HVS 3×/week for 31 days neither showed overt signs of toxicity nor significant body weight changes. These findings suggest the potential of HVS to exhibit preferential anticancer effects in clinically relevant mouse model without notable toxicity unlike most targeted cancer therapies. Overall, HVS suppressed its target *in vivo* in the xenografted human breast tumor tissue and selectively decreased cancer cell proliferation at doses that are well tolerated and thus, prevented the progression of invasive breast cancer. The *in vivo* data reinforced the *in vitro* cellular conclusions and confirmed c-Met as the primary target of the antiproliferative effects of HVS, allowing for potential future use of this lead compound to control c-Met-dependent malignancies.

## MATERIALS AND METHODS

### Chemicals, reagents, and antibodies

All chemicals were purchased from Sigma-Aldrich (St. Louis, MO), unless otherwise stated. *S*-Oleocanthal was isolated from EVOO (Daily Chef, batch number: L022RE-565, Italy). All antibodies were purchased from Cell Signaling Technologies (Beverly, MA) and used at a dilution of 1:1,000, unless otherwise stated. Antibody for Brk was acquired from Abnova (Walnut, CA). Antibody for p-Brk was obtained from Santa Cruz Biotechnology (Santa Cruz, CA). The α-tubulin antibody was obtained from NeoMarkers (Freemont, CA) and used at 1:20,000. Goat anti-rabbit and goat anti-mouse secondary antibodies were purchased from Perkin Elmer Biosciences (Boston, MA). Growth factors used in cell assays, including HGF and EGF, were acquired from PeproTech Inc. (Rocky Hill, NJ). Wild-type c-Met recombinant human protein and its oncogenic mutant variants were purchased from Life Sciences.

### Chemical synthesis of HVS by chemoselective Mitsunobu esterification reaction

Triphenylphosphine (TPP, 280 mg, 1 mM) was added in portions to a freshly prepared solution of homovanillyl alcohol (1 mmol) and sinapic acid (1 mmol equivalent) in anhydrous tetrahydrofuran (THF, 3.5 mL) at 0°C. Diisopropylazodicarboxylate (DIAD, 208 μL, 1 mmol) was then added dropwise to the mixture. The reaction mixture was then stirred at 0°C for 30 min. The mixture was then warmed and stirred for 48 h at rt [34]. The reaction was monitored till completion by thin layer chromatography. The reaction mixture was worked up by removal of the solvent under reduced pressure, saturated solution of sodium bicarbonate (10 mL) was added, and then the mixture was extracted with ethyl acetate (3 × 20 mL). The combined organic layers were washed with brine, dried over anhydrous sodium sulfate, filtered, and the filtrate was evaporated under reduced pressure. The crude product was collected and purified by column chromatography on Sephadex LH-20 using dichloromethane to remove the Mitsunobu byproducts followed by normal phase silica gel 60 column chromatography, eluted with gradient *n*-hexane-ethyl acetate system, to afford HVS (Supplementary Material).

### Cell lines and culture conditions

Unless otherwise mentioned, human cancer cell lines were purchased from the American Type Culture Collection (ATCC, Manassas, VA). MDA-MB-231/GFP human breast carcinoma cells were acquired from Cell Biolabs, Inc. (San Diego, CA). All cancer cells were maintained in RPMI-1640 supplemented with 10% fetal bovine serum (FBS), 100 U/mL penicillin G, 100 μg/mL streptomycin and 2 mmol/L glutamine. The immortalized non-tumorigenic human mammary epithelial MCF-10A cell line was purchased from ATCC. MCF-10A cells were cultured in DMEM/F12 supplemented with 5% horse serum, 0.5 μg/mL hydrocortisone, 20 ng/mL EGF, 100 U/mL penicillin G, 100 ng/mL cholera toxin, 100 μg/mL streptomycin, and 10 μg/mL insulin. Cells were subcultured upon attaining 80% confluency. All cells were maintained at 37°C under 5% CO_2_ in humidified incubator, using standard cell culture techniques. A stock solution was prepared by dissolving HVS in sterilized DMSO at a concentration of 10 mM for all assays. Working solutions at their final concentrations for each assay were prepared in appropriate culture medium immediately prior to use. The vehicle (DMSO) control was prepared by adding the maximum volume of DMSO, used in preparing HVS, to the appropriate media type such that the final DMSO concentration was maintained as the same in all treatment groups within a given experiment and never exceeded 0.1%. *S* (−)-Oleocanthal was used in all experiments as a positive control at doses selected based on earlier studies [19].

### Biochemical kinase assay

The Z’-LYTE™ Kinase Assay-Tyr6 Peptide kit (Life Sciences) was used to assess the ability of HVS to inhibit the catalytic activity of wild-type c-Met (Product# PV3143) and its oncogenic variant M1250T (Product# PV3968). Briefly, 20 μL/well reactions were set up in 96-well plates containing kinase buffer, 200 μM ATP, 4 μM Z-LYTE™ Tyr6 Peptide substrate, 2500 ng mL^−1^ c-Met kinase and compound of interest as an inhibitor. After 1 h incubation at rt, 10 μL development solution containing site-specific protease was added to each well. Incubation was continued for 1 h. The reaction was then stopped, and the fluorescent signal ratio of 445 nm (coumarin)/520 nm (fluorescein) was determined on a plate reader (BioTek FLx800™), which reflects the peptide substrate cleavage status and/or the kinase inhibitory activity in the reaction. The inhibition rate (%) was calculated using the following equation: % Inhibition = 100 - [(Activity of enzyme with tested compound - Min)/ (Max - Min)] × 100 (Max: the enzyme activity measured in the presence of enzyme, substrates, and cofactors; Min: the enzyme activity in the presence of substrates, cofactors and in the absence of enzyme).

### Measurement of viable cell number

Viable cell count was determined using the MTT colorimetric assay. The optical density of each sample was measured at 570 nm on a microplate reader (BioTek, VT). The number of cells per well was calculated using a standard curve prepared at the start of each experiment by plating various numbers of cells (1,000–60,000 cells/well), as determined by a hemocytometer.

### Cell growth and viability studies

MDA-MB-231, MDA-MB-468, MCF-7, BT-474, and T-47D cells in exponential growth, were seeded at a density of 1 × 10^4^ cells per well (6 wells/group) in 96-well culture plates and maintained in RPMI-1640 media supplemented with 10% FBS and allowed to adhere overnight at 37°C under 5% CO_2_. The next day, cells were washed with phosphate buffer saline (PBS), divided into different treatment groups and then fed various concentrations of HVS in serum-free media containing 40 ng/mL HGF (which induced maximum growth after 72 h) or no HGF (0.5% FBS was added to maintain the viability of the cells throughout the experiment). Cells in all groups were fed fresh treatment media every other day for a 72 h treatment period. Viable cell number was determined using the MTT assay. Control and treatment media were replaced with fresh media, and 50 μL fresh MTT solution (1 mg mL^−1^) was added to each well and plates were re-incubated for 4 h at 37°C. The color reaction was stopped by removing the media and adding 100 μL DMSO in each well to dissolve the formed formazan crystals. Incubation at 37°C was resumed for up to 20 minutes to ensure complete dissolution of crystals. Absorbance was determined at λ 570 nm using an ELISA plate microreader (BioTek, VT). The % cell survival was calculated as follows: % survival = (Cell No._treatment_/Cell No._DMSO_) × 100.

The effect of HVS on the proliferation and growth of immortalized non-tumorigenic mammary epithelial cells MCF10A were evaluated by plating these cells at a density of 1 × 10^4^ cells per well (6 wells/group) in 96- well culture plates, maintained in DMEM/F12 media containing 5% horse serum, and allowed to attach overnight. The next day, cells were washed with PBS, divided into different treatment groups and then treated with various concentrations of HVS in serum-free defined media containing 40 ng/mL HGF, and incubation resumed at 37°C under 5% CO_2_ for 24 h. Viable cell number was determined using the MTT assay.

### LDH cytotoxicity assay

The Cayman's LDH cytotoxicity assay was accomplished in compliance with manufacturer procedures after optimization regarding the number of cells per well and serum concentration used [59]. Briefly, the MDA-MB-231 cells were seeded into a 96-well plate at a density of 3 × 10^4^ cells/well suspended in 200 μL culture medium. The same culture medium volume was added into three wells as a background control. After cell recovery and attachment, media were removed and cells were treated with 200 μL of 5% serum supplemented media containing HVS at the desired concentrations in triplicates. Triton X-100 (20 μL, 10%) solution was added to three wells containing only the cells (maximum release) and 20 μL of assay buffer to other three cell-free wells (spontaneous release). The microplate was then incubated under 5% CO_2_ at 37°C for 24 h. Plate was then centrifuged at 400 × g for five minutes and 100 μL of cell supernatant from each well was transferred to a new 96-well plate. Reaction buffer (100 μL of NAD^+^, lactic acid, iodonitrotetrazolium (INT), reconstituted diaphorase) was added to each well and the plate was incubated with gentle shaking for 30 min at rt. Finally, absorbance was measured at 490 nm using Synergy 2 microplate reader (BioTek, Vermont). The percent cytotoxicity was calculated using the following formula:

$$\%\text{Cytotoxicity}=[\frac{\left(\text{Experimental value A}490)-(\text{Spontaneous release A}490\right)}{\left(\text{Maximum release A}490)-(\text{Spontaneous release A}490\right)}]\times 100$$

### Wound-healing assay

The *in vitro* wound-healing assay was used to assess directional cell motility in two dimensions. MDA-MB-231 cells were plated in sterile 24-well plates (6 replicates/group) and allowed to form a confluent monolayer per well overnight. Wounds were then inflicted in each cell monolayer using a sterile 200 μL pipette tip. The media was removed and cells were washed twice with PBS and once with fresh RPMI medium to remove cell debris. Cells were then incubated with HVS at the desired concentrations in serum-free defined media containing 40 ng/mL HGF. Cells were incubated for 24 h and afterward, media was removed and cells were washed with pre-cooled PBS, fixed with methanol previously cooled to −20°C, and stained with Giemsa. Wound healing was visualized at 0 and 24 h by Nikon ECLIPSE TE200-U microscope and digital images were captured using Nikon NIS Elements software (Nikon Instruments Inc., Melville, NY). The distance traveled by the cells was determined by measuring the wound width at 24 h and subtracting it from the wound width at the start of treatment (*t* ^0^, zero time). The values obtained were then expressed as % migration, setting the gap width at *t* ^0^ as 100%. The distance migrated was calculated in three or more randomly selected fields per treatment group.

### Cell invasion assay

The Cultrex^®^ BME cell invasion assay (Trevigen, Inc., Gaithersburg, MD) was conducted according to the vendor's provided protocol. The top invasion chamber inserts were coated with 50 μL/well of 1× BME solution and incubated overnight at 37°C under 5% CO_2_. The coating solution was then aspirated off and 50,000/50 μL of MDA-MB-231 cells suspended in fresh RPMI-1640 medium were then seeded to each well at the top chamber. HVS was prepared, in serum-free medium supplemented with 40 ng/mL HGF, at 6× the desired concentrations and 10 μL of each tested concentration was added in triplicate to the wells of the top chamber to achieve the final test concentrations (1, 5, 10 and 20 μM). About 150 μL of RPMI-1640 medium, containing 10% FBS and penicillin/streptomycin as well as fibronectin (1 μL/mL) and *N*-formyl-Met-Leu-Phe (10 nM) as chemoattractants, was then added to each well in the lower chamber. Plates were gathered and re-incubated at 37°C under 5% CO_2_ for 24 h after which both chambers were carefully aspirated and washed with 100 μL/well washing buffer supplemented with the kit. About 100 μL of cell dissociation/calcein-AM solution was added to each well in the bottom chamber and the plate was incubated at 37°C under 5% CO_2_ for 1 h. The cells internalize calcein-AM, and the intracellular esterases cleave the acetomethyl ester (AM) moiety to generate free calcein. Fluorescence of samples was measured at λ_excitation_ 485 nm and λ_emission_ 528 nm using an ELISA plate reader (BioTek, VT). Relative fluorescence units (RFU) were used to calculate the number of cells invaded through the BME coat using a standard curve prepared by plating various numbers of cells prior to the experiment. Mean percent invasion of different treatments was calculated in relative to the DMSO-treated control wells.

### Cell scattering assay

DU145 human prostate cancer cells were seeded into a 24-well plate at a density of 4 × 10^4^ cells/well and grown in serum-containing medium overnight. After cell recovery and attachment, cells were pre-treated with designated concentrations of HVS for 30 min in serum-free media. Either 33 ng/mL HGF or 100 ng/mL EGF was then spiked into the appropriate wells and cells were allowed to scatter while incubating the plates at 37°C under 5% CO_2_ for 16 h. Cells were then fixed with 4% paraformaldehyde for 15 min at rt and stained with phalloidin (Molecular Probes, Carlsbad, CA). Cells were imaged using a Nikon Eclipse TE300 inverted epifluorescence microscope and CoolSnap fx digital camera with IPLab software. Images were pseudo-colored using Image J and representative 10× images are shown in Figure 5C.

### 3D spheroid assay

MDA-MB-231 or DU145 cells grown in 10% FBS DMEM were used. Cells were collected after trypsinization and resuspended in phenol-red free DMEM supplemented with 10% FBS. Cells were then labeled with CellTracker Red (Life Technologies) for 5 min. Cells were washed with PBS after labeling and seeded into 96-well Corning 7007 ultra-low attachment (ULA) round bottom plates at a density of 1,000 cells/well suspended in 100 μL phenol-red free media, supplemented with 10% FBS and 5% Matrigel. This protocol resulted in the formation of a single spheroid in the center of each well of identical size, after 24 h. Spheroids were supplemented either with DMSO as vehicle control or designated concentrations of HVS or *S*-oleocanthal. Spheroids were incubated at 37°C under 5% CO_2_ in IncuCyte ZOOM real time imaging system (Essen Bioscience) and images were automatically acquired every 4 h for 72 h post-treatment. The software associated with the IncuCyte was designed to identify the red object in each well and calculate the average area of each spheroid. The data were expressed as fold increase in spheroids size at the end of the experiment using the average red object area in each well. The assay was repeated twice and performed with 8 spheroids per treatment group.

### Western blot analysis

Western blot analysis was conducted according to the previously described method [19]. The whole breast cancer cell extracts were prepared in RIPA buffer (Qiagen Sciences Inc., Valencia, CA). The whole DU145 prostate cancer cell lysates were taken directly in boiling Laemmli buffer and boiled for five minutes. Protein concentration was determined using the BCA assay (Bio-Rad Laboratories, Hercules, CA) according to the manufacturer's instructions. Equivalent amounts of protein were electrophoresed on SDS-polyacrylamide gels under reducing conditions. The gels were then electroblotted onto PVDF membranes. These PVDF membranes were then blocked with 2% BSA in 10 mM Tris-HCl containing 50 mM NaCl and 0.1% Tween 20, pH 7.4 (TBST) and then, probed with the indicated specific primary antibodies overnight at 4°C. Membranes were then extensively washed with TBST and incubated with respective horseradish peroxidase-conjugated anti-rabbit or anti-mouse secondary antibodies in 2% BSA in TBST for 1 h at rt followed by rinsing with TBST for 5 times. Blots were then visualized using an enhanced chemiluminescence detection system according to the manufacturer's instructions (Pierce, Rockford, IL). Images of protein bands from all treatment groups within a given experiment were acquired using Kodak Gel Logic 1500 Imaging System (Carestream Health Inc, New Haven, CT). Pierce ECL 2 Western blotting Substrate (Life Technologies, Carlsbad, CA) was used for chemiluminescent detection on X-ray film (Phenix, Candler, NC) for DU145 cells. The visualization of α- or β-tubulin was used to ensure equal sample loading in each lane. All experiments were repeated at least three times and representative Western blot images from each experiment are shown in Figures 7 and 8.

### c-Met phosphorylation and downstream c-Met-dependent signaling pathways

MDA-MB-231, MCF-7 and BT-474 human breast cancer cells in addition to DU145 prostate cancer cells were used to assess the effect of HVS treatment on c-Met phosphorylation and c-Met-dependent signaling pathways. Cells were initially plated at a density of 1 × 10^6^ cells/100 mm culture plates in RPMI-1640 media supplemented with 10% FBS and allowed to adhere overnight. Breast cancer cells were then washed twice with 1× PBS and incubated in the respective control or treatment media containing 0.5% FBS for 72 h before being stimulated with 100 ng/mL human recombinant HGF for 10 min before cell lysis. DU145 cells were treated with a varying range of concentrations of HVS or *S* (−)-oleocanthal for 30 min and then stimulated with HGF for additional 30 min before cell lysis. Phosphorylation and expression levels of c-Met protein and its downstream signaling molecules Akt, MAPK, FAK, Brk, paxillin, and Rac1 were assessed by Western blot probed with the indicated specific antibodies.

### Molecular modeling

The *in silico* experiments were carried out using Schrödinger molecular modeling software package (Supplementary Material).

### Binding site evaluation using SiteMap

This evaluation uses site-point groups and energetic properties of grid points to evaluate the binding sites. A number of properties are computed for the three tested kinases (ABL1, c-Met, and IGF1R), some of which are used in the final site scoring, including the volume and the size of each binding pocket. These properties are calculated as previously described [33].

### Kinase profiling

HVS at 10 μM was screened against a panel of 15 human kinases using Life Sciences SelectScreen^®^ Kinase Profiling Selectivity Testing Services (Life Sciences, Carlsbad, CA). The percent inhibition for each kinase was determined by a time-resolved fluorescence resonance energy transfer assay. Protocols are available at http://www.thermofisher.com/us/en/home/products-and-services/services/custom-services/screening-and-profiling-services/selectscreen-profiling-service/selectscreen-kinase-profiling-service.html?CID=fl-we113108.

### *In vivo* studies

#### Animals

Female athymic nude mice (Foxn1^nu^/Foxn^1+^, 4–5 weeks old) were purchased from Harlan (Indianapolis, IN). The animals were acclimated to the animal housing facility and maintained under clean room conditions in sterile filter top cages with Alpha-Dri bedding and housed on high efficiency particulate air-filtered ventilated racks at a temperature of 18–25°C, with a relative humidity of 55 to 65% and a 12 h light/dark cycle, for at least one week before the study. The mice had free access to drinking water and pelleted rodent chow (no. 7012, Harlan/Teklad, Madison, WI). All animal experiments were approved by the Institutional Animal Care and Use Committee, University of Louisiana at Monroe, and were handled in strict accordance with good animal practice as defined by the NIH guidelines.

#### Xenograft model in athymic mice

MDA-MB-231/GFP human breast cancer cells were harvested, pelleted by centrifugation at 850 × g for 5 minutes, and resuspended in sterile serum-free DMEM medium (20 μL). Xylazine (1.0 mL xylazine at 20 mg/mL) was added to 10 mL ketamine at 100 mg/mL to make 11.0 mL at 92 mg/mL of stock. About 1.0 mL of this solution was diluted with 9.0 mL sterile normal saline to make a 9.2 mg/mL solution. About 10 mL of this solution/kg was used for anesthesia, which is equivalent to 10 μL/g. Tumor cell suspension (1 × 10^6^ cells/20 μL) was inoculated subcutaneously into the second mammary gland fat pad just beneath the nipple of each animal after anesthesia to generate orthotopic breast tumors.

#### Efficacy studies

Efficacy studies were done in athymic mice bearing MDA-MB-231 tumor xenografts to determine the effect of HVS on tumor growth. At 48 h post-inoculation, mice were randomly divided into two groups: (i) the vehicle-treated control group (*n* = 5) and (ii) the HVS-treated group (*n* = 5). Treatment (3 ×/week) started 5 days post-inoculation with intraperitoneal (i.p.) administered vehicle control (DMSO/saline) or 10 mg/kg of HVS for four cycles. Selection of this dose was based on earlier preliminary *in vivo* studies on HVS. HVS treatment was prepared by dissolving 5 mg of HVS in 100 μL DMSO to prepare a stock solution, then dissolving 40 μL of stock solution in 960 μL normal saline just prior to the injection. The mice were monitored by measuring tumor volume, body weight, and clinical observations. Tumor volume (V) was calculated using the formula *V* = (L × W^2^)/2, where L is the length in mm and W is the width in mm of tumors as measured using a digital Vernier caliper every other day. Results are expressed on indicated days as the mean tumor volume ± SEM indicated for groups of mice. Percent (%) TGI was measured on the last day of study for drug-treated compared with vehicle-treated mice. % TGI is calculated as 100 × {1-[(Treated_Final day_ -Treated_Day 1_)/ (Control_Final day_- Control_Day 1_)]}. To assess differences in tumor size between the treated versus the control groups, One-Way ANOVA analysis of variance was performed and significance was defined as *P* < 0.05. The body weight of each mouse was monitored every other day, and expressed as the mean ± SEM. All mice were sacrificed on the 31st day post-inoculation, and individual tumors were excised and weighed. Breast tumor tissues were stored at −80°C until total protein extraction for Western blot analysis or stored in 70% ethanol at rt for immunohistochemistry studies.

#### c-Met pharmacodynamics (PD) study

The PD studies were conducted in athymic nude mice bearing MDA-MB-231 tumor xenografts by determining the effects of HVS on c-Met signaling. Mice were humanely euthanized at designated times following HVS administration, and resected breast tumor tissues were stored at −80°C until protein extracted. Breast tumor tissues were homogenized in RIPA buffer (Qiagen Sciences Inc., CA) using an electric homogenizer. Protein lysates were generated, and protein concentrations were determined using the BCA assay (Bio-Rad Laboratories, CA). The levels of total and phosphorylated c-Met were determined using Western blot analysis. The visualization of β-tubulin was used to ensure equal sample loading in each lane. The experiment was repeated three times and representative Western blot images are shown in Figure 10D.

#### Immunohistochemistry

Athymic mice with established MDA-MB-231 human breast tumor xenografts were given HVS (10 mg/kg) or vehicle control. Breast tumor tissues were dissected 4 h after dosing and were formalin-fixed for 24 h before being transferred to 70% ethanol. Subsequently, tumor samples were processed for immunohistochemistry analysis of 6-μm-thick sections in accordance to a standard protocol that was run on an automated Sakura Prisma unit. The tumor specimens were processed with the use of alcohols and xylene and then infiltrated in paraffin wax using the Excelsior™ ES Tissue Processor. Paraffin sections were dewaxed in xylene, rinsed in alcohol, rehydrated in water and then placed in citric buffer (PH 6.0) and treated in a microwave oven with high power for 3 min and 10% Goat serum for 30 min and then, baked onto microscope slides. Subsequently, primary antibodies with proper dilution were applied on the sections as follows: CD31 (Pierce Product# PA5-32321; 1:50 dilution, 1 h at rt) and Ki-67 (Cell Signaling Product# #9027; 1:150 dilution, 1 h at rt). Secondary antibodies (Ventana Multimer Anti-Rb-HRP Product #760-4311; 24 minutes at rt) were then applied. Signals were developed with Vector ImmPACT DAB Product#SK-4105 for 8 minutes at rt. All immunostained sections were finally counterstained using hematoxylin solution for 1 min at rt.

#### Determination of positive Ki-67 cells and microvessel density

To evaluate positive Ki-67 cells in breast cancer tissues, five areas were examined at a magnification of ×200. MVD of breast tumor tissue sections was evaluated. Any CD-31 positively stained endothelial cell or endothelial cell cluster was counted as one microvessel. The mean microvessel count of the five most vascular areas was taken as the MVD, which was expressed as the absolute number of microvessels per 1.485 mm^2^ (×200 field).

#### Statistics

The results are presented as the means ± SEM of at least three independent experiments. Differences among various treatment groups were determined by ANOVA followed by Dunnett's test using PASW statistics^®^ version 18 (Quarry Bay, Hong Kong). A difference of *p* < 0.05 was considered statistically significant compared to the vehicle-treated control group. The IC_50_ values were determined using a non-linear regression curve fitting analysis using GraphPad Prism software version 6 (La Jolla, CA).

## SUPPLEMENTARY MATERIALS FIGURES AND TABLES



## Abbreviations

HGF: hepatocyte growth factor
c-Met: mesenchymal-epithelial transition factor
EGF: epidermal growth factor
3D: three-dimensional
ABL1: abelson murine leukemia viral oncogene homolog 1
TNBC: triple negative breast cancer
RTK: receptor tyrosine kinase
EGFR: epidermal growth factor receptor
BRAF: v-RAF murine sarcoma viral oncogene homolog B1
NSCLC: non-small cell lung cancer
ROS1: v-ros avian UR2 sarcoma virus oncogene homolog 1
ALK: anaplastic lymphoma kinase
VEGFR2: vascular endothelial growth factor receptor 2
EVOO: extra-virgin olive oil
IC_50_: 50% inhibitory concentration
ATP: adenosine triphosphate
PDB: protein data bank
Å: angstrom
HB: hydrogen bond
MTT: 3-[45-dimethylthiazol-2-yl]-25-diphenyl-tetrazolium bromide
ERα: estrogen receptor alpha
LDH: lactate dehydrogenase
BME: basement membrane extract
DMSO: dimethyl sulfoxide
2D: two-dimensional
Rac1: Ras-related C3 botulinum toxin substrate 1
Cdc42: cell division control protein 42 homolog
PI3K: phosphoinositide 3-kinase
Akt: protein kinase B
Brk: breast tumor kinase
Erk: extracellular signal regulated kinase
MAPK: mitogen-activated protein kinase
FAK: focal adhesion kinase
TK: tyrosine kinase
RON: recepteur d'origine nantais
TYRO3: tyrosine protein kinase
IGF1R: insulin-like growth factor receptor 1
Kit: v-kit Hardy-Zuckerman 4 feline sarcoma viral oncogene homolog
XP: extra-precision
PD: pharmacodynamics
TGI: tumor growth inhibition
GFP: green fluorescent protein
i.p.: intraperitoneal
MVD: microvessel density
Ret: rearranged during transfection
HER2: human epidermal growth factor receptor 2
NFκB: nuclear factor κB
Rock: Rho-associated coiled-coil-containing protein kinase
Crk: creatine kinase
P130Cas: P130 Crk-associated substrate
ABL2: abelson murine leukemia viral oncogene homolog 2
P53: tumor suppressor protein
TPP: triphenylphosphine
THF: tetrahydrofuran
DIAD: diisopropyl-azodicarboxylate
rt: room temperature
ATCC: American type culture collection
RPMI: Roswell park memorial institute
FBS: fetal bovine serum
DMEM/F12: Dulbecco›s modified eagle medium: nutrient mixture F-12
PBS: phosphate buffer saline
ELISA: enzyme-linked immunosorbent assay
NAD: nicotinamide adenine dinucleotide
INT: iodonitrotetrazolium
AM: acetomethyl ester
RFU: relative fluorescence unit
ULA: ultra-low attachment
RIPA: radioimmunoprecipitation assay
BCA: bicinchoninic acid assay
SDS: sodium dodecyl sulfate
PVDF: polyvinylidene fluoride
BSA: bovine serum albumin
TBST: Tris-buffered saline with Tween 20
NIH: National Institutes of Health
SEM: standard error of a mean
ANOVA: analysis of variance
Anti-Rb-HRP: anti-rabbit horseradish peroxidase-conjugated
DAB: 33′-diamino-benzidine
ErbB: erythroblastic leukemia viral oncogene homolog
HER4: human epidermal growth factor receptor 4
FGFR1: fibroblast growth factor receptor 1
Flt-1: Fms-like tyrosine kinase
KDR: kinase insert domain receptor
MST1R: macrophage stimulating 1 receptor
PDGFRβ: platelet-derived growth factor receptor β
RSE: receptor sectatoris.
