# Supplementary material for: The oleocanthal-based homovanillyl sinapate as a novel c-Met inhibitor
Source: Oncotarget. 2016 Apr 11;7(22):32247–73. doi: 10.18632/oncotarget.8681 (PMC5078011; doi:10.18632/oncotarget.8681)
Supplement: Supplementary file 1 [file oncotarget-07-32247-s001.pdf]

The oleocanthal-based homovanillyl sinapate as a novel c-Met inhibitor

Supplementary Materials

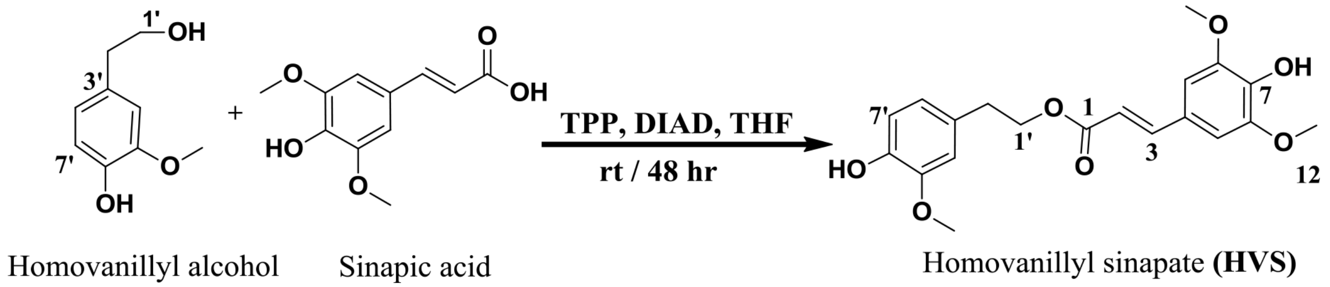

Scheme S1: Synthesis of homovanillyl sinapate (HVS). *(E)-4-hydroxy-3-methoxyphenethyl 3-(4-hydroxy-3,5-dimethoxyphenyl) acrylate* (HVS).

HVS was prepared in 60% yield by the reaction of 168 mg of homovanillyl alcohol with 224 mg of sinapic acid. White amorphous powder; ^{1}H and ^{13}C NMR, see Table S1 and Figures S1 and S2; HRESIMS m/z 373.1285 $[M-H]^-$ (calcd for $C_{20}H_{21}O_7$ 373.1287).^{1}H and ^{13}C NMR spectra of HVS were recorded in deuterated chloroform ($CDCl_3$), using tetramethylsilane (TMS) as an internal standard, on a JEOL Eclipse-ECS NMR spectrometer operating at 400 MHz for ^{1}H NMR and 100 MHz for ^{13}C NMR. Chemical shifts (δ) are reported in ppm and coupling constants (*J*) in Hz (Table S1). Data are reported as follows: chemical shift, multiplicity (s = singlet, *d* = doublet, *t* = triplet, *dd* = doublet of doublet), and coupling constants. High-resolution ESIMS experiments were conducted using a JEOL JMS-T100 LP AccuTOF LC-Plus, equipped with an ESI source (JEOL Co. Ltd., Tokyo, Japan). ESI-MS detection was set using negative ion mode; needle voltage set at -2,000 V; and the ring lens and orifice 1 and 2 voltages set at -10, -35, and -7 V, respectively. Nitrogen was used as the nebulizing and desolvation gas, and pressure was maintained constant at 0.608 MPa. Desolvation chamber and orifice 1 temperatures were set to 250°C and 120°C, respectively. Results were obtained using Mass Center software, MS-56010MP (JEOL).

Supplementary Table S1: ^{1}H NMR and ^{13}C NMR Spectroscopic Data of HVS

Position	HVS	
	δ_H, mult. (J in Hz)[a]	δ_C[b]
1	-	167.3, qC
2	6.27, d (16.0)	115.8, CH
3	7.56, d (16.0)	145.3, CH
4	-	125.9, qC
5	6.73, s	105.1, CH
6	-	147.3, qC
7	-	137.2, qC
8	-	147.3, qC
9	6.73, s	105.1, CH
10	3.89, s	56.4, CH_3
11	3.89, s	56.4, CH_3
12	3.85, s	56.0, CH_3
1′	4.36, t (7.1)	65.2, CH_2
2′	2.92, t (7.1)	35.0, CH_2
3′	-	129.7, qC
4′	6.74, d (2.3)	111.5, CH
5′	-	146.6, qC
6′	-	144.4 qC
7′	6.84, d (8.7)	114.5, CH
8′	6.73, dd (8.7, 2.3)	121.7, CH

[a]In $CDCl_3$, 400 MHz for ^{1}H NMR. Coupling constants (J) in Hz.
[b]In $CDCl_3$, 100 MHz for ^{13}C NMR. Carbon multiplicities were determined by PENDANT experiment, qC = quaternary, CH = methine, CH_2 = methylene, CH_3 = methyl carbons.

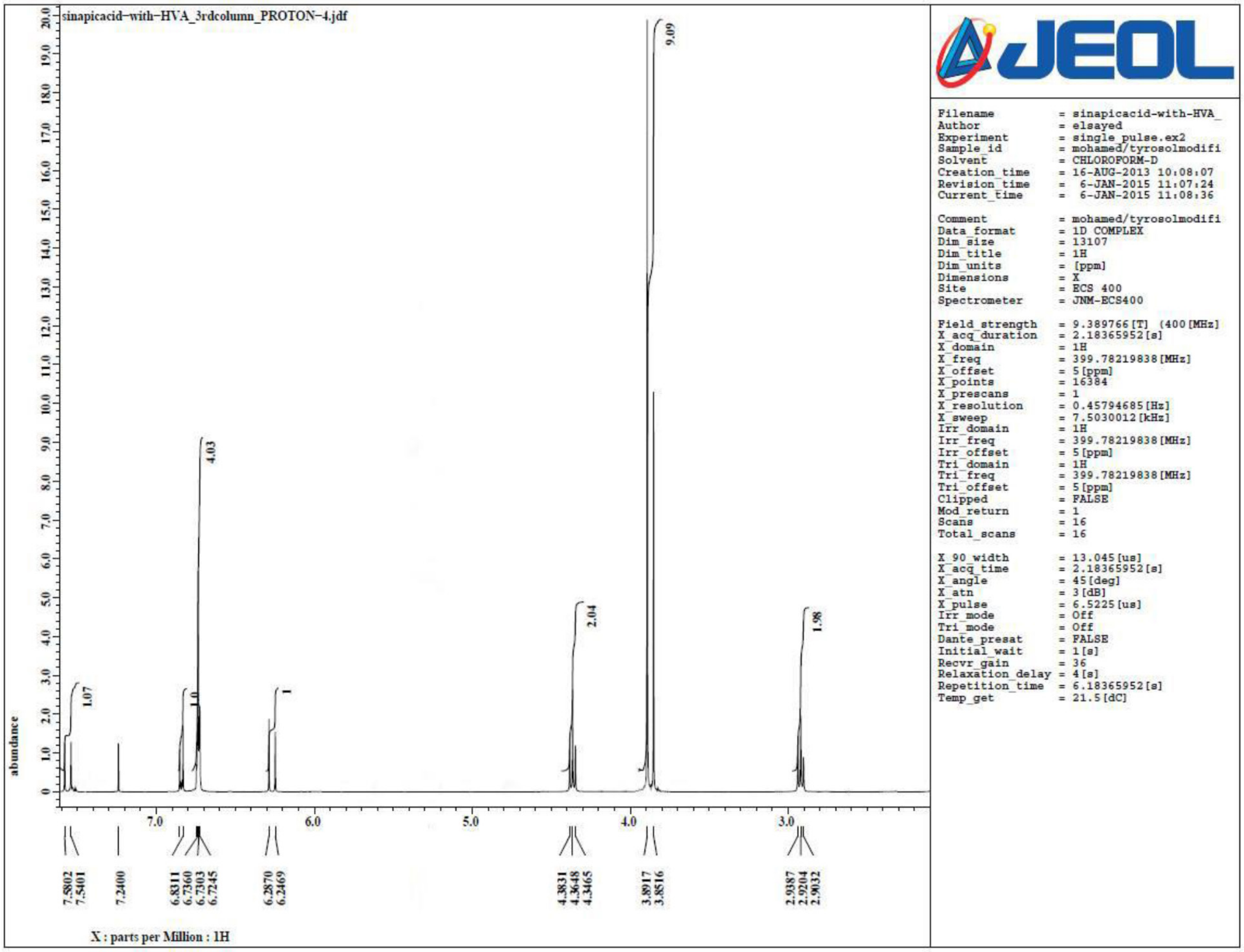

Supplementary Figure S1: ^{1}H NMR Spectrum of HVS.

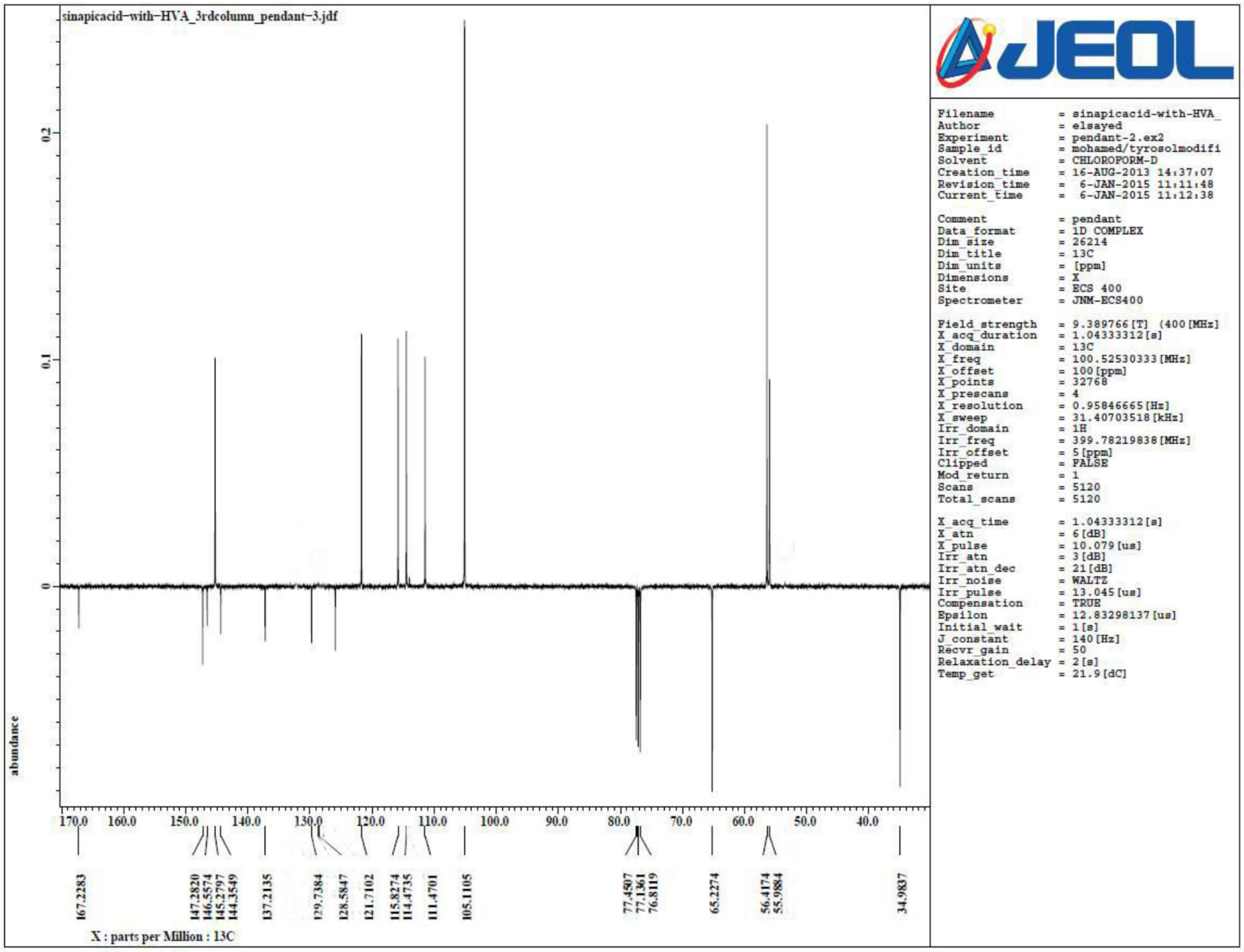

Supplementary Figure S2: ^{13}C NMR (PENDANT) Spectrum of HVS.

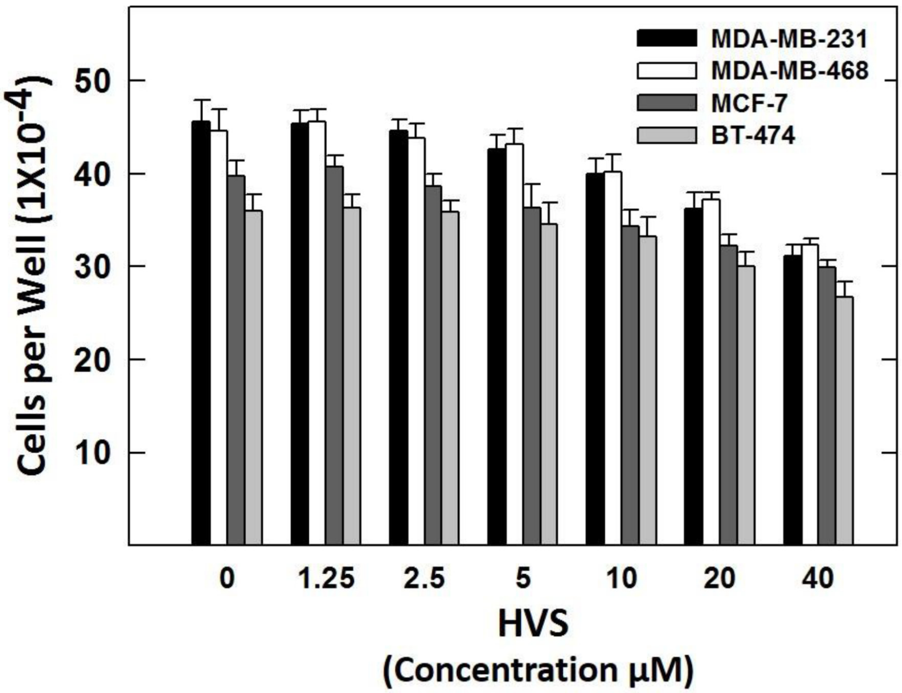

Supplementary Figure S3: Effect of HVS treatment on the growth of MDA-MB-231, MDA-MB-468, MCF-7, and BT-474 breast cancer cells, in the absence of HGF and using 0.5% FBS, after 72 h treatment period. Viable cell count was determined using MTT assay. Vertical bars indicate the mean cell count ± SEM of *n* = 3 in each treatment group.

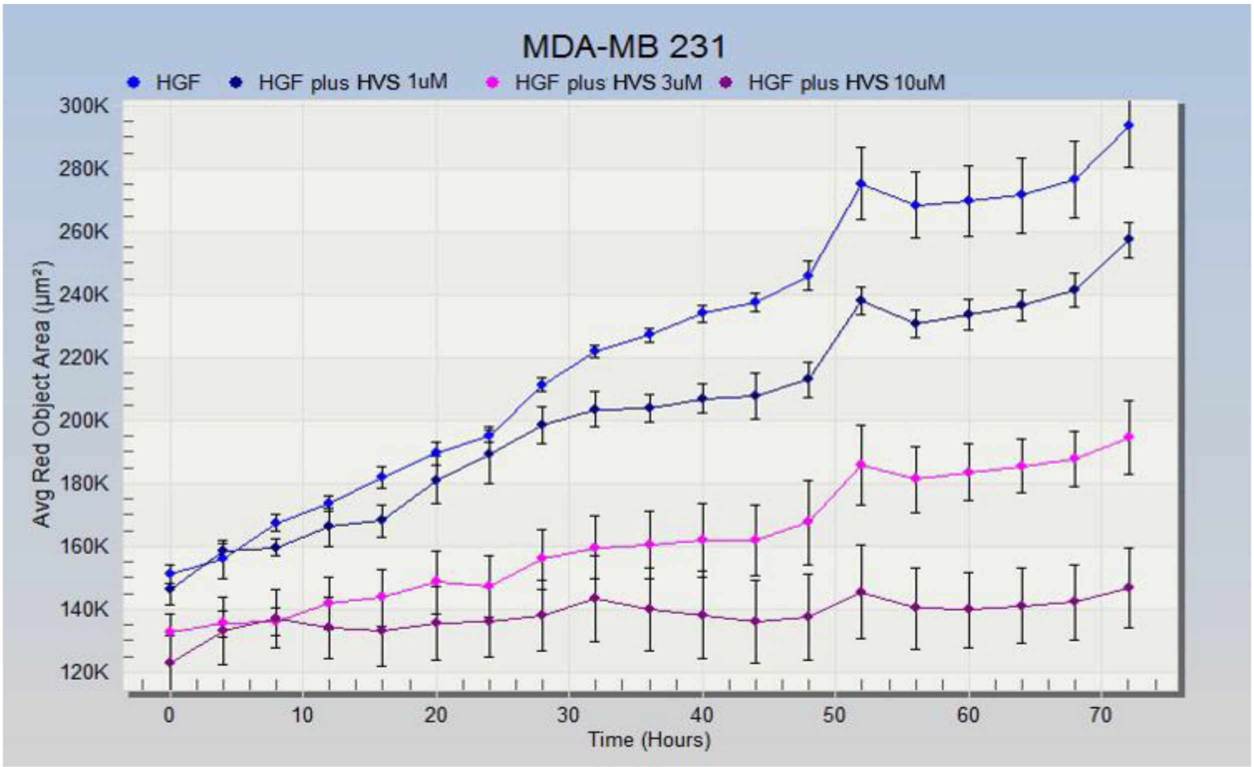

Supplementary Figure S4: MDA-MD231 spheroids treated with HGF and/or different concentrations of HVS for 72 h.

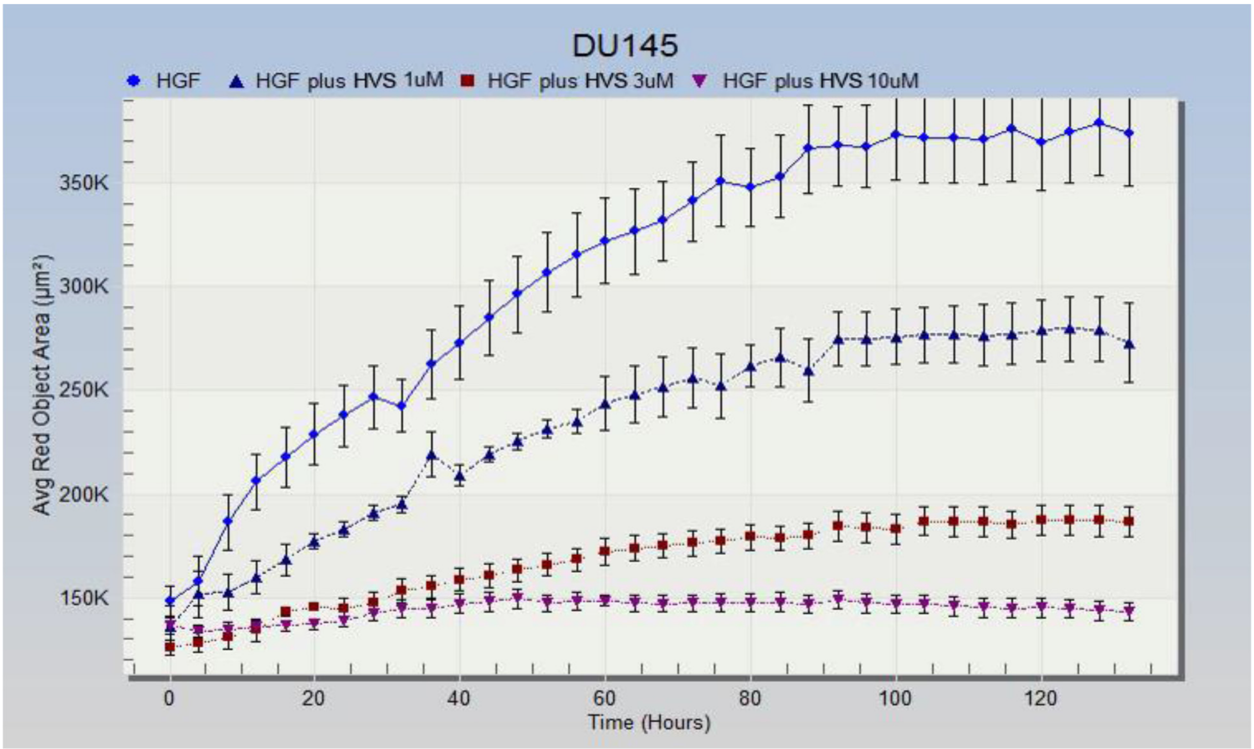

Supplementary Figure S5: DU145 spheroids treated with HGF and/or different concentrations of HVS for 72 h.

Molecular modeling experimental

The *in silico* experiments were carried out using Schrödinger molecular modeling software package installed on an iMac 27-inch Z0PG workstation with a 3.5 GHz Quad-core Intel Core i7, Turbo Boost up to 3.9 GHz, processor and 16 GB RAM (Apple, Cupertino, CA).

Protein structure preparation

Two X-ray crystal structures of c-Met tyrosine kinase domain; PDB codes: 4XYF [1] and 2RFS [2] of the wild- and mutant-types, respectively, were retrieved from the Protein Data Bank (www.rcsb.org). In order to identify the structural basis which has contributed to differences in affinity among c-Met, ABL1 and IGF1R, the crystal structures of the ABL1 (PDB code: 3OXZ [3]) and IGF1R (PDB code: 1JQH [4]) have been also obtained from the Protein Data Bank. The Protein Preparation Wizard was implemented to prepare the kinase domain of each protein. The protein was reprocessed by assigning bond orders, adding hydrogens, creating disulfide bonds and optimizing H-bonding networks using PROPKA (Jensen Research Group, Denmark). Finally, energy minimization with RMSD value of 0.30°A was applied using an Optimized Potentials for Liquid Simulation (OPLS_2005, Schrödinger) force field.

Ligand structure preparation

The chemical structure of HVS was sketched on the Maestro 9.3 panel interface (Maestro, version 9.3, 2012, Schrödinger). The LigPrep 2.3 module (LigPrep, version 2.3, 2012, Schrödinger) was implemented to generate the 3D structure and to search for different conformers. The OPLS (OPLS_2005, Schrödinger) force field was applied to geometrically optimize the ligand structure and to compute partial atomic charges. Finally, 32 poses per ligand were generated with different steric features for subsequent docking studies.

Molecular docking

The prepared X-ray crystal structures of each protein were used to generate receptor energy grids applying the default value of the protein atomic scale (1.0°A) within the cubic box centered on the co-crystallized ligand of each crystal structure. HVS was then docked using the Glide 5.8 module (Glide, version 5.8, 2012, Schrödinger) in extra-precision (XP) mode. Modeling scores were generated using the Glide-Dock program's empirical scoring functions.

REFERENCES

1. Peterson EA, Teffera Y, Albrecht BK, Bauer D, Bellon SF, Boezio A, Boezio C, Broome MA, Choquette D, Copeland KW, Dussault I, Lewis R, Lin MH, et al. Discovery of potent and selective 8-fluorotriazolopyridine c-met inhibitors. J Med Chem. 2015; 58:2417–2430.
2. Bellon SF, Kaplan-Lefko P, Yang Y, Zhang Y, Moriguchi J, Rex K, Johnson CW, Rose PE, Long AM, O'Connor AB, Gu Y, Coxon A, Kim TS, et al. C-met inhibitors with novel binding mode show activity against several hereditary papillary renal cell carcinoma-related mutations. J Biol Chem. 2008; 283:2675–2683.
3. Zhou T, Commodore L, Huang WS, Wang Y, Thomas M, Keats J, Xu Q, Rivera VM, Shakespeare WC, Clackson T, Dalgarno DC, Zhu X. Structural mechanism of the pan-bcr-abl inhibitor ponatinib (ap24534): Lessons for overcoming kinase inhibitor resistance. Chem Biol Drug Des. 2011; 77:1–11.
4. Pautsch A, Zoephel A, Ahorn H, Spevak W, Hauptmann R, Nar H. Crystal structure of bisphosphorylated igf-1 receptor kinase: Insight into domain movements upon kinase activation. Structure. 2001; 9:955–965.
